# Supplementary material for: The theoretical understanding of risk perception and dual-method contraceptive decision-making among Texas adolescent and young adult cisgender females
Source: PLoS One. 2026 Apr 1;21(4):e0343687. doi: 10.1371/journal.pone.0343687 (PMC13042665; doi:10.1371/journal.pone.0343687)
Supplement: Attachment — Submitted filename: PONE-D-24-49168_R1 ME Sep 6.pdf [file pone.0343687.s004.pdf]

PLOS ONE

The Theoretical Understanding of Risk Perception and Dual-Method Contraceptive Decision-Making among Texas Adolescent and Young Adult Cisgender Females

--Manuscript Draft--

**Manuscript Number:**	PONE-D-24-49168R1
**Article Type:**	Research Article
**Full Title:**	The Theoretical Understanding of Risk Perception and Dual-Method Contraceptive Decision-Making among Texas Adolescent and Young Adult Cisgender Females
**Short Title:**	Risk Perception and Dual-Method Contraceptive Decision-Making among Adolescent and Young Adult Women
**Corresponding Author:**	Lauren Holt, Ph.D., RN<br>Duke University School of Nursing<br>Durham, NC UNITED STATES OF AMERICA
**Keywords:**	contraception; Dual-method; decision-making; Risk perception; Adolescent women; Young adult women; women's health; grounded theory; sexually transmitted infection
**Abstract:**	In this paper we use a constructivist grounded theory approach to conduct individual, qualitative interviews with a racially and ethnically diverse group of adolescent and young adult cisgender females (AYACF) (18 to 24 years of age) to generate theoretical knowledge of the relationship between Social Determinants of Health (SDOH), STI and HIV risk perception, and dual-method contraceptive decision-making (the use of, both, a prescribed contraceptive [commonly referred to as “birth control”] and a condom during vaginal intercourse). Twenty-five AYACF were selected through theoretical sampling. Data were collected using semi-structured interviews and analyzed using constant comparison. AYACF expressed having a greater fear of pregnancy than sexually transmitted infections (STIs). They described deferring to their partners and making assumptions about their partner’s STI status based on partner and relationship factors, therefore not needing to engage in condom negotiation.Mother-daughter sexual health conversations and state school-based sex education and abortion policies influenced participants’ STI risk perception and dual-method contraceptive decision-making. Participants who were diagnosed with an STI reported experiencing a shift in perception about their likelihood of acquiring an STI, often resulting in behavior change. These findings are important as they provide insight into the complex decision-making processes, influenced by SDOH, that a racially and ethnically diverse group of AYACF associate with STI risk perception and dual-method contraceptive use.
**Order of Authors:**	Lauren Holt, Ph.D., RN
	Amy Corneli, PhD
	Eleanor Stevenson, PhD, RN, FAAN
	Jonas Swartz, MD, MPH
	Michael Relf, PhD, RN, ACNS-BC, CNE, FAAN
**Response to Reviewers:**	Dear editors of PLOS ONE,<br><br>Thank you for the opportunity to revise our manuscript, “The Theoretical Understanding of Risk Perception and Dual-Method Contraceptive Decision-Making among Texas Adolescent and Young Adult Women” (PONE-D-24-49168).<br><br>We have provided responses to each of the questions from the two reviewers below.<br><br>We would be happy to provide any additional information that may be needed.
**Additional Information:**	
**Question**	**Response**
**Financial Disclosure**	Yes

Powered by Editorial Manager® and ProduXion Manager® from Aries Systems Corporation

Enter a financial disclosure statement that describes the sources of funding for the work included in this submission. Review the submission guidelines for detailed requirements. View published research articles from *PLOS ONE* for specific examples.<br><br>This statement is required for submission and **will appear in the published article** if the submission is accepted. Please make sure it is accurate.<br><br>**Funded studies**<br>Enter a statement with the following details:<br>• Initials of the authors who received each award<br>• Grant numbers awarded to each author<br>• The full name of each funder<br>• URL of each funder website<br>• Did the sponsors or funders play any role in the study design, data collection and analysis, decision to publish, or preparation of the manuscript?<br><br>Did you receive funding for this work?	
Please add funding details.<br>as follow-up to "**Financial Disclosure**<br><br>Enter a financial disclosure statement that describes the sources of funding for the work included in this submission. Review the submission guidelines for detailed requirements. View published research articles from *PLOS ONE* for specific examples.<br><br>This statement is required for submission and **will appear in the published article** if the submission is accepted. Please make sure it is accurate.	LH<br>-Grant number: 383000723 (Sigma Theta Tau/Association of Nurses in AIDS Care)<br>-Funders: Sigma Theta Tau/Association of Nurses in AIDS Care; Margolis Scholars Program<br>-Website for Sigma: https://www.sigmanursing.org/advance-elevate/research/research-grants/sigma-theta-tau-international-association-of-nurses-in-aids-care-grant<br>-Website for Margolis:<br>https://healthpolicy.duke.edu/scholars#:~:text=The%20Margolis%20Scholars%20program%20is%20a%20selective%20program,well%20as%20leadership%20potential%20to%20improve%20health%20policy.<br>-No, the funders did not play any role in the study design, data collection and analysis, decision to publish, or preparation of the manuscript

Powered by Editorial Manager® and ProduXion Manager® from Aries Systems Corporation

**Funded studies**<br>Enter a statement with the following details:<br>• Initials of the authors who received each award<br>• Grant numbers awarded to each author<br>• The full name of each funder<br>• URL of each funder website<br>• Did the sponsors or funders play any role in the study design, data collection and analysis, decision to publish, or preparation of the manuscript?<br><br>Did you receive funding for this work?"	
Please select the country of your main research funder (please select carefully as in some cases this is used in fee calculation).<br>as follow-up to "**Financial Disclosure**<br><br>Enter a financial disclosure statement that describes the sources of funding for the work included in this submission. Review the submission guidelines for detailed requirements. View published research articles from *PLOS ONE* for specific examples.<br><br>This statement is required for submission and **will appear in the published article** if the submission is accepted. Please make sure it is accurate.<br><br>**Funded studies**<br>Enter a statement with the following details:<br>• Initials of the authors who received each award<br>• Grant numbers awarded to each author<br>• The full name of each funder<br>• URL of each funder website<br>• Did the sponsors or funders play any role in the study design, data collection and analysis, decision to publish, or preparation of the manuscript?<br><br>Did you receive funding for this work?"	UNITED STATES - US
**Competing Interests**	The authors have declared that no competing interests exist.

Powered by Editorial Manager® and ProduXion Manager® from Aries Systems Corporation

Use the instructions below to enter a competing interest statement for this submission. On behalf of all authors, disclose any competing interests that could be perceived to bias this work—acknowledging all financial support and any other relevant financial or non-financial competing interests.<br><br>This statement is required for submission and **will appear in the published article** if the submission is accepted. Please make sure it is accurate and that any funding sources listed in your Funding Information later in the submission form are also declared in your Financial Disclosure statement.<br><br>View published research articles from *PLOS ONE* for specific examples.<br><br>**NO authors have competing interests**<br><br>Enter: *The authors have declared that no competing interests exist.*<br><br>**Authors with competing interests**<br><br>Enter competing interest details beginning with this statement:<br><br>*I have read the journal's policy and the authors of this manuscript have the following competing interests: [insert competing interests here]*<br><br>* typeset	
**Ethics Statement**<br><br>Enter an ethics statement for this submission. This statement is required if the study involved:<br><br>• Human participants<br>• Human specimens or tissue<br>• Vertebrate animals or cephalopods<br>• Vertebrate embryos or tissues	-Duke University Institutional Review Board<br>-Pro00111285<br>-Oral consent was obtained from all participants

Powered by Editorial Manager® and ProduXion Manager® from Aries Systems Corporation

• Field research<br><br>Write "N/A" if the submission does not require an ethics statement.<br><br>General guidance is provided below. Consult the submission guidelines for detailed instructions. **Make sure that all information entered here is included in the Methods section of the manuscript.**	

**Format for specific study types**<br><br>**Human Subject Research (involving human participants and/or tissue)**<br>• Give the name of the institutional review board or ethics committee that approved the study<br>• Include the approval number and/or a statement indicating approval of this research<br>• Indicate the form of consent obtained (written/oral) or the reason that consent was not obtained (e.g. the data were analyzed anonymously)<br><br>**Animal Research (involving vertebrate animals, embryos or tissues)**<br>• Provide the name of the Institutional Animal Care and Use Committee (IACUC) or other relevant ethics board that reviewed the study protocol, and indicate whether they approved this research or granted a formal waiver of ethical approval<br>• Include an approval number if one was obtained<br>• If the study involved *non-human primates*, add *additional details* about animal welfare and steps taken to ameliorate suffering<br>• If anesthesia, euthanasia, or any kind of animal sacrifice is part of the study, include briefly which substances and/or methods were applied<br><br>**Field Research**<br><br>Include the following details if this study involves the collection of plant, animal, or other materials from a natural setting:<br>• Field permit number<br>• Name of the institution or relevant body that granted permission	
**Data Availability**<br><br>Authors are required to make all data underlying the findings described fully available, without restriction, and from the time of publication. PLOS allows rare exceptions to address legal and ethical concerns. See the PLOS Data Policy and FAQ for detailed information.	No - some restrictions will apply

Powered by Editorial Manager® and ProduXion Manager® from Aries Systems Corporation

<table>
<tr>
<td>A Data Availability Statement describing where the data can be found is required at submission. Your answers to this question constitute the Data Availability Statement and will be published in the article, if accepted.

Important: Stating ‘data available on request from the author’ is not sufficient. If your data are only available upon request, select ‘No’ for the first question and explain your exceptional situation in the text box.

Do the authors confirm that all data underlying the findings described in their manuscript are fully available without restriction?</td>
<td></td>
</tr>
<tr>
<td>Describe where the data may be found in full sentences. If you are copying our sample text, replace any instances of XXX with the appropriate details.

• If the data are held or will be held in a public repository, include URLs, accession numbers or DOIs. If this information will only be available after acceptance, indicate this by ticking the box below. For example: All XXX files are available from the XXX database (accession number(s) XXX, XXX.).
• If the data are all contained within the manuscript and/or Supporting Information files, enter the following: All relevant data are within the manuscript and its Supporting Information files.
• If neither of these applies but you are able to provide details of access elsewhere, with or without limitations, please do so. For example:

Data cannot be shared publicly because of [XXX]. Data are available from the XXX Institutional Data Access / Ethics Committee (contact via XXX) for researchers who meet the criteria for access to confidential data.

The data underlying the results presented in the study are available from (include the name of the third party</td>
<td>Data cannot be shared publicly or with other researchers outside of those involved with the study due to the sensitive nature of the topic and the need to maintain participant privacy.Further, it was not indicated to participants that the de-identified data would be made publicly available at the conclusion of the study. As such, because the participants were not made aware that this would happen, the data cannot be made publicly available at this time as imposed by Duke IRB. Data requests can be made to
Duke IRB
Suite 900 Erwin Square
2200 West Main Street
Campus Box # 104026
Durham, NC 27705

Phone: (919) 668-5111

Email:ResearchServiceDesk@duke.edu</td>
</tr>
</table>

Powered by Editorial Manager® and ProduXion Manager® from Aries Systems Corporation

and contact information or URL).<br>• This text is appropriate if the data are owned by a third party and authors do not have permission to share the data.<br><br>* typeset	
Additional data availability information:	

Powered by Editorial Manager® and ProduXion Manager® from Aries Systems Corporation

Manuscript

Click here to access/download;Manuscript;Manuscript_DualMethodContracep

The Theoretical Understanding of Risk Perception and Dual-Method Contraceptive Decision-Making among Texas Adolescent and Young Adult Cisgender Females

Lauren Holt, PhD, MSN, RN[a], Amy Corneli, PhD, MPH[b], Eleanor Stevenson, PhD, RN, FAAN[a], Jonas Swartz, MD, MPH[c], and Michael Relf, PhD, RN, ACNS-BC, CNE, FAAN[a]

[a] School of Nursing, Duke University School of Nursing, 307 Trent Drive, Durham, NC 27710; lauren.holt@duke.edu, eleanor.stevenson@duke.edu, michael.relf@duke.edu

[b] Department of Population Health Sciences, 215 Morris Street, Suite 210, Durham, NC 27701; amy.corneli@duke.edu

[c] Department of Obstetrics & Gynecology, Duke University Medical Center, 201 Trent Drive, 203 Baker House, Durham, NC 27710; jonas.swartz@duke.edu

Corresponding author:

Lauren Holt, PHD, RN

1310 Karen Ave.

Austin, TX 78757

P 325.650.8282

Lauren.holt@duke.edu

1. Introduction

Sexually transmitted infections (STIs), including HIV, are a significant public health concern. In Texas, STIs are on the rise as primary syphilis rates doubled over the last five years, and congenital syphilis rates are at an all-time high (1). Adolescent and young adult cisgender females (AYACF) between 18 and 24 years old have experienced a consistent increase in syphilis over the last five years, while accounting for higher rates of chlamydia than any age or gender group (1).

Despite rising STI rates, pregnancy rates have steadily declined among Texas AYACF (2) a feat largely attributed to an increase in access and utilization of prescribed contraceptive methods methods (i.e intrauterine devices (IUDs), birth control pills, hormonal implants, injections, patches, and rings) (2-4). Although prescribed contraceptives are 90-99% effective effective in reducing the risk of pregnancy when used correctly (5), they do not protect against STIs or HIV.

The risk of acquiring an STI and HIV can be reduced through correct and consistent condom use during oral, vaginal, and anal sex (6). However, only 23% of AYACF use condoms during every sexual encounter (7). AYACF who use prescribed contraceptives are less likely to practice dual-method contraceptive use (8, 9), which occurs when a prescribed contraceptive and a condom are both used during intercourse (10). Further, adolescent females who use long-acting reversible contraceptives (LARCs) such as IUDs and implants are less likely to practice dual-method contraceptive use and have more sexual partners than those using short-acting reversible contraceptives (SARCs) such as oral birth control pills (11-14) (15).

Condom is already a dual protection method. It needs to be considered throughout the work.

Increasing trends in STI rates and limited dual-method contraceptive use highlight the need to investigate attitudes and beliefs related to contraceptive use and STI and HIV risk perception among AYACF. Risk perception analysis is an important factor in dual-method contraceptive decision-making as AYACF with a high STI and HIV risk perception are more likely to initiate dual-method contraceptive use than those who perceive themselves as low risk (12, 16-18). While risk perception analysis has been identified as an integral part of decision-making (19), the processes associated with STI and HIV risk perception analysis and dual-method contraceptive decision-making among AYACF are poorly understood.

Contraceptive method selection is based on a checklist.
Based on some question, client will decid…

Dual-method contraceptive use is more likely to occur in women with a high STI and HIV risk perception than those who perceive themselves as low risk (16). Further, women with low STI and HIV risk perception are more likely to engage in high-risk sexual behaviors (12, 17, 18). Previous studies have shown risk perception to be a determinant factor for engaging in STI and HIV protective behaviors (20-22). Risk perception plays an integral role in the decision-making process (19).However, the processes associated with risk perception and dual-method contraceptive decision-making are poorly understood. Moreover, as professional associations and practice guidelines continue to promote LARCs as the first-line contraceptive method among adolescent women (23, 24), more research is needed to understand how the utilization of prescribed contraceptives impacts one's perceived risk of acquiring an STI and the processes associated with dual-method contraceptive use decision making.

It will be good to have a look on WHo guidelines like MEC.

4.1.1. STI and HIV Disparities

AYACF belonging to structurally oppressed racial and ethnic groups are disproportionately more affected by STIs than White AYACF. For example, Black females are five times more likely to be diagnosed with chlamydia and three times more likely to be diagnosed with HIV

Type of sexual relationship is among the checlist questions.

when compared to White females, while Hispanic females are twice as likely to be diagnosed with syphilis (25). Moreover, American Indian/Alaskan Native females are six times more likely to be diagnosed with gonorrhea, and five times more likely to be diagnosed with syphilis than their White counterparts (25). The existing age, gender, racial, and ethnic STI and HIV disparities highlight the need to consider how social determinants influence dual-method contraceptive use among AYACF.

The World Health Organization (WHO) defines the SDOH as the circumstances under which individuals are born, raised, live, work, and age, and the available systems to deal with illness (26). These circumstances are shaped by structural determinants which encompass social and political mechanisms that influence individual and collective access to resources, further contributing to social class division (27). Structural determinants such as statewide policies pertaining to sexual and reproductive health access and education, and societal and cultural norms and values influence dual-method contraceptive use among AYACF, resulting in STI and HIV disparities (28-30).

Since Roe V. Wade was overturned in June 2022, abortion because a state's right issue, and thus became illegal in twelve states, including Texas, causing cisgender women and people with uteruses (and cisgender men) to re-evaluate their pregnancy prevention methods. For example, over the last two years LARC use has increased, and the number of tubal sterilization and vasectomy procedures increased among young adults ages 18 to 30 years old (31, 32). Additionally, the use of emergency contraception (commonly referred to as Plan B) surged after June 2022 (and has since subsided) while oral contraceptives have decreased in states where abortion laws are the most restrictive (33).

So, it seems family planning guidleines are not the main reason for increase of LARC use.

Based on this text, rules and regulation changes, especially for abortion, had a significant impact in this regard.

Sex education also plays a major role in dual-method contraceptive decision-making. Currently, only 18 states require sex education content to be medically accurate and only 27 states require HIV education to be included in sex education curricula (34). Further, only 20 states are required to provide students with information on contraceptive methods (34). As a result, only 52% of U.S. youth receive education about where to obtain prescribed contraceptive methods and only 59% are educated on condom application (28). While there is a strong correlation between inadequate sexual health education policy and climbing STI and HIV rates, societal and cultural norms and values also play an integral role in determining STI and HIV risk and disparities.

Societal and cultural norms and values are structural which determine STI and HIV risk and can inadvertently serve as a barrier to engaging in dual-method contraceptive use, if not acknowledged (29, 35, 36). Gendered societal expectations and sexual stereotypes are the foundation of uneven relationship dynamics, resulting in the decreased use of condoms and/or prescribed contraceptives (29, 30, 37, 38). Further, gendered societal expectations and sexual stereotypes are disproportionately experienced by AYACF belonging to structurally oppressed racial and ethnic groups, contributing to glaring STI and HIV disparities (29).

In the context of SDOH, structural determinants operate through intermediary determinants which directly affect dual-method contraceptive use among AYACF (27). Health care access and quality play a critical role in determining dual-method contraceptive use as AYACF with access to health insurance are more likely to engage in dual-method contraceptive use and less likely to report an STI and HIV than those who are medically uninsured (39, 40). Additionally, psychosocial factors such as the interpersonal relationship dynamics between an AYACF and her partner determine dual-method contraceptive use as AYACF who report higher levels of

relationship trust and commitment are less likely to practice dual-method contraceptive use than those in less trusting or committed relationships (12, 41). Meanwhile, negative relationship factors such as violence and conflict are associated with inconsistent, or lack of, dual-method contraceptive use (42, 43). To understand the processes associated with dual-method contraceptive decision-making and STI and HIV risk perception among AYACF, healthcare access, and quality and relationship factors must be taken into consideration.

4.1.2. Study Purpose

The overall objective of this qualitative study was to gain insight into STI and HIV-related health factors among a racially and ethnically diverse AYACF population who are using prescribed contraceptives. Using a grounded theory approach, we conducted individual qualitative interviews with sexually active AYACF using prescribed contraceptives to generate theoretical knowledge of the relationship between SDOH, STI and HIV risk perception, and decision-making and use that knoweldge to inform the development of a conceptual model for future theoretical testing and intervention development. Here we report on the following two aims of the larger study:

Aim 1: Understand AYACF's STI and HIV risk perception and decision-making related to dual-method contraceptive use.

Aim 2: Develop a conceptual model explaining the processes associated with risk perception analysis and decision-making related to dual-method contraceptive use among sexually active AYACF using prescribed contraceptives.

4.2. Methods

4.2.1. Study Design

This study implemented a constructivist grounded theory qualitative design to generate a middle-range theory related to STI and HIV risk perception analysis and dual-method contraception decision-making among sexually active AYACF (44). Following constructivist grounded theory procedures, the proposed study integrated the essential components by 1) theoretically sampling participants to get representativeness of the population, 2) conducting in-depth intensive interviews, 3) developing and using memos to ensure dependability and confirmability in increasing the trustworthiness of the data and findings and, 4) using a constant comparison approach in data analysis (44). The primary data collection strategy was intensive individual semi-structured qualitative interviews collected at one point in time to provide an in-depth exploration of the participants' experiences and situations (44).

4.2.2. Participants and Setting

In conducting a constructivist grounded theory study, it was imperative to select participants who had first-hand experience that would address the aims of the proposed study (44). To understand the processes of STI and HIV risk perception and dual-method contraceptive decision-making among sexually active AYACF using prescribed contraceptives, participants had to meet the following inclusion criteria: 1) self-identify as a cisgender female; 2) be 18 to 24 years old; 3) report vaginal intercourse in the last 12 months; and 4) currently using a prescribed contraceptive method such as LARCs (i.e. IUD's, implants) or SARCs (i.e. contraceptive pills, injections, patches, rings).

Does it mean that condom not prescribed for anybody?
Why?

Why female condom use was not assessed?

I want to know whether one health clininc can cover all people or just a limited area?

If it covers a limited area, then whether it can be rersesntative for that district?

Which types of social/ economi/ political features were prominent in the clinic area?

Study recruitment occurred from 11/01/2023 to 30/11/2023 at a health clinic in urban Texas that provides free gynecological, birth control, family planning, and STI screening and treatment services to adolescents and young adults in one of the largest and most racially and ethnically diverse counties in the US (45, 46). Further, the county where the health clinic resides reports the highest STI case numbers in the state of Texas while accounting for 1 in 4 new HIV diagnoses at a rate that is almost twice the state average (47).

4.2.3. Data Collection

A semi-structured interview format was appropriate for this study as it facilitated data collection, while still allowing participants to freely discuss thoughts or ideas that might not have been considered by the research team (48). Interview questions were drafted by the research team who specialize in sexual and reproductive health to ensure content validity. Further, participant input contributed to the development of additional questions that were asked in future interviews. After the fourteenth interview, triangulation was used to identify new questions to add to the interview guide that would elicit further discussion about perceived monogamy and STI risk. While searching for qualitative studies that focused on condom use among adolescents and young adults, the research team identified a study by Bolton, McKay (49) and adapted questions from their interview guide starting with the fifteenth interview. Based on observations from the first fourteen interviews, the research team added questions such as "How did you know you were/are in a monogamous relationship?"," What was happening in the relationship at the time?" and "What do you think is the likelihood that you will acquire an STI?" (49). Key questions that guided the interviews are presented in **Table 1**.

Table. 1. Social Determinants of Health Interview Guide

Rapport-building questions:<br>• Tell me a little about yourself.<br>• What interested you in this study?
Structural Determinants<br><br>STI/HIV Prevention *(Sex Education Policy/Societal and Cultural Norms and Values)*<br>Question 1: As previously mentioned in the screening, this is a study for sexually active women who are using prescribed contraceptives, commonly referred to as birth control, and we are interested in learning how they perceive STI/HIV risk. What do you think are the most important ways a young woman can protect herself against pregnancy and sexually transmitted infections (STIs) including HIV?<br>• Probe: Tell me about your experience in learning about prescribed contraceptive and condom use.<br>• Probe: Tell me about your sexual health education experience in school<br>• Probe: Can you share with me how women in your community protect themselves from pregnancy and STIs, including HIV.<br>• *Probe: Tell me about how one's culture might influence using a birth control and/or condom during sex*<br>• Probe: How might where someone grew up influence their understanding of sexual health and prescribed birth control
Intermediary Determinants<br><br>Prescribed Contraceptives *(Health System and Psychosocial)*<br>Question 2: Could you tell me about the events that led to your decision to seek prescribed contraceptive?<br>• Probe: As you thought about seeking prescribed contraceptives, who were the individuals you talked to for guidance?<br>• Probe: Can you share with me the information you were considering before starting a prescribed contraceptive?<br>• Probe: Tell me about any barriers you faced in obtaining your prescribed contraceptive?<br>• Probe: Tell me about your interaction with your provider during the visit you were prescribed contraceptive<br>• Probe: Tell me about the things you were thinking about when you made the decision to start a specific prescribed contraceptive.<br><br>Prescribed Contraceptive and Condom Use *(Psychosocial)*<br>Question 3: How would you describe how you viewed condom use before you got on a prescribed contraceptive?<br>• Probe: What pregnancy prevention methods did you use before starting a prescribed contraceptive?

• Probe: What contributed to your decision to use (or not to use) condoms before starting a prescribed contraceptive?<br>• Probe: Could you tell me about how your views towards condom use may have changed since starting a prescribed contraceptive?<br><br>Sexual Partners *(Psychosocial)*<br>Question 4: Since we have been discussing condom use, I would like to learn about your current or most recent sexual partner.<br>• Probe: Can you share with me conversations that you and your partner had regarding sexually transmitted infection (STI) prevention?<br>• Probe: Can you tell me how you both came to this understanding?<br><br>For participants who are currently in or had previously been in a monogamous relationship:<br>• *How did you know you were/are in a monogamous relationship?[153]<br>• *Probe: What was happening in the relationship at that time?[153]<br><br>For all participants:<br>• *What do you think is the likelihood that you will acquire an STI?[153]
Closing: Is there anything else you would like to share with me that we haven't talked about today?<br>• Before we end our conversation, I wanted to give you an opportunity to ask any questions.<br><br>*Questions inserted after fourteenth interview

Before interviews, each participant completed a demographic data form which included age, sex, gender identity, race and ethnicity information, relationship status, and type of prescribed contraception in use. Individual semi-structured interviews took place virtually via Zoom and were recorded with an encrypted audio recording device. All interviews were transcribed verbatim by a transcription service and checked against the recording for accuracy. Investigator memos were written immediately following the interviews and supplemented throughout the data analysis process to capture thoughts and develop connections (44). For participating in the study, each participant was compensated with a $40 Amazon gift card.

4.2.4. Study Procedures

Theoretical sampling was implemented based on age, race, ethnicity, and type of prescribed contraceptive (SARC vs. LARC) (44). All selected participants were recruited in person at the health clinic or referred to the research team by other participants. Once participants agreed to the interview, they were provided with a consent form to be signed before the interview. A total of twenty-five one-time, semi-structured interviews were conducted via Zoom after obtaining informed consent. All twenty-five interviews were conducted by one member of the research team (LH) who has training in qualitative research methods. Following theoretical sampling procedures (44), after one interview was completed, transcribed, and analyzed, the next participant was theoretically recruited and selected for an interview to ensure the representativeness of cisgender adolescent young adult women's experiences. Characteristics of previous participants (i.e. age, race, ethnicity, education, birth control method) were components considered in the theoretical sampling process. This sampling approach helped to ensure the confirmability of diverse experiences.

4.2.5. Data Analysis

An inductive qualitative content analysis, using constant comparison, was conducted to explain the processes associated with risk perception analysis and decision-making related to dual-method contraceptive use among sexually active AYACF using prescribed contraceptives (44). The transcribed verbatim transcripts were imported into NVivo for coding and analysis (50). Constant comparison analysis was performed between two members of the research team who have experience in qualitative data analysis and sexual and reproductive health research throughout the length of the data collection period and during three phases of coding- initial, focused, and theoretical. To add precision and clarity to the data analysis, the research team

conducted theoretical coding by 1) analyzing and synthesizing relationships between focused codes and 2) building a conceptual model explaining the processes associated with risk perception analysis and decision-making related to dual-method contraceptive use among sexually active AYACF using prescribed contraceptives (44, 51). The development of the conceptual model began during the theoretical coding phase of the first interview and occurred throughout the theoretical coding phase of the remaining interviews.

4.2.6. Trustworthiness and Rigor

To ensure credibility, triangulation was used to compare study findings to the literature throughout the data analysis process. Over the course of the study, data, procedures, and tools were constantly compared against the literature to assess the need for alterations. To ensure confirmability, reflexive journaling was conducted after each interview to capture thoughts and perceptions and identify any potential biases. To ensure dependability, an audit trail was kept detailing the developmental and implementation stages of this study.

4.3. Results

As demonstrated in **Table 2**, a total of twenty-five AYACF were recruited for this study; the mean age of participants was 20.7 years and represented diverse racial (African American, 56%; White, 40%; Asian, 2%; Unknown, 2%) and ethnic identities (Hispanic, 48%; Non-Hispanic, 48%; Unknown, 2%). The participants were mainly college-educated (80%) without children (88%) and currently in a monogamous relationship with one male partner (52%). Additionally, the majority of participants had previously been diagnosed with an STI (52%) and were currently using Depo Provera (40%) as their prescribed contraceptive method. Interviews ranged from 18 to 55 minutes, with an average of 38 minutes per interview.

Table 2: Participant Demographics

Age	#	%
18 years old	2	8%
19 years old	3	12%
20 years old	11	44%
21 years old	2	8%
22 years old	2	8%
23 years old	1	4%
24 years old	4	16%

Race/Ethnicity	#	%
White	1	4%
Black	11	44%
Hispanic	8	32%
Asian	1	4%
Other	4	16%

Education	#	%
Current College Student	19	76%
Graduated College	1	4%
Non-College	4	16%
High School	1	4%

Previous STI Diagnosis	#	%
Yes	13	52%
No	12	48%

Birth Control Method	#	%
Shot	10	40%
Implant	5	20%
Pill	8	32%
Ring	1	4%
Patch	1	4%

Relationship Status	#	%
Single and having casual sex with one male partner	8	32%
Single and having casual sex with more than one male partner	4	16%
In monogamous relationship with one male partner	13	52%
In open relationship with more than one male partner	0	0%

The process of STI risk perception analysis and dual-method contraceptive decision-making consists of the following categories 1) Fear of pregnancy greater than fear of STIs, 2) Making assumptions and partner deferral, and 3) STI Diagnosis: A new reality (**Figure 1. Dual Method Contraception Decision-Making Factors**). For AYACF, SDOH and personal background perpetuate the belief that pregnancy is a more significant concern than STIs. As AYACF navigate sexual relationships, they make assumptions about a sexual partner's STI status based on specific partner characteristics and relationship dynamics. Together, relationship dynamics and partner characteristics influence the decision to defer to the sexual partner for dual-method contraceptive decision-making. Additionally, a general lack of concern for STIs contributed to the decision to forgo condom use and rely on STI testing after engaging in sexual intercourse without a condom. As a result of deferring to the sexual partner and substituting condom use for STI testing, an STI diagnosis occurred, causing a shift in STI risk perception, which at times resulted in a behavior change.

4.3.1. Fear of Pregnancy > Fear of STIs

For many participants, the fear of an unwanted pregnancy was far greater than their fear of acquiring an STI. Although all participants were prescribed contraceptive users, many reported feeling *scared* and *anxious* about getting pregnant. For example, a twenty-one-year-old Asian college student compared her fear of pregnancy to a feeling of *impending doom*. While the fear of pregnancy was palpable among participants, their fear of STIs was minimal, playing a significant role in their STI risk perception. A twenty-year-old Hispanic college student shared her perspective on pregnancy and STIs:

> *Well, I think pregnancy is definitely a lifetime, like a for-life commitment. And that's definitely way scarier, even though STIs are scary for sure. I don't know. I just feel like*

when you think of pregnancy, it's like really scary. Like, really scary, I mean, especially for women. I've always just been scared of my life stopping there.

Having an overwhelming fear of unwanted pregnancy contributed to dual-method contraceptive decision-making as several participants stopped using condoms once initiating a prescribed contraceptive. For example, a twenty-year-old Hispanic college student with a previous STI diagnosis described her experience of using condoms before initiating prescribed contraceptive use:

So I have used a condom since, like, I started the first time [being sexually active]. *And I've been using a condom since then until I went on birth control for the whole time, and then I stopped using a condom.*

Further, participants who engaged in dual-method contraceptive use reported using condoms for additional pregnancy protection as opposed to STI and HIV protection. A twenty-four-year-old Hispanic college student explained why she used condoms in addition to a prescribed contraceptive.

I think it was mostly, like, my thinking of condoms was, like, me just not getting pregnant. It was, like, another barrier for birth control, basically.

4.3.2. Social Determinants of Health

Recent changes to Texas abortion laws exacerbated participants' fear of unwanted pregnancy. As a result of newly implemented state laws banning abortion, participants considered additional ways to prevent pregnancy and detect pregnancy at the earliest stage possible. In the quote below, a nineteen-year-old African American college student shared her experience:

I feel like this makes me more conscious of knowing, like, I need to know right now if I think I'm pregnant because we should not be wasting time.

Further, participants contemplated how they could access or self-induce an abortion in the instance that they became pregnant. For example, a nineteen-year-old college student who identified as Hispanic, African American, and White shared how she would approach an unplanned pregnancy.

I'm pretty confident that I would catch it [pregnancy] *in time, and I will just get mailed in some abortion pills and just take that and naturally miscarry.*

A twenty-one-year-old Asian college student shared a similar perspective on what she would do if she were to experience an unplanned pregnancy.

I can't afford an abortion. Do I have to buy a bunch of vitamin C? To have like one of those, like self-abortions or whatever I hear people talking about like a hanger.

School-based sex education also played a significant role in how participants perceived pregnancy and STIs. For example, a twenty-one-year-old Asian college student shared her sex education experience:

They just instill a lot of fear of not getting pregnant at an early age and not getting STDs. And it's, it's effective because I'm deadly scared of getting pregnant at my age.

The majority of participants received a sexual health education in school that primarily focused on abstinence. A nineteen-year-old African American college student with a prior STI diagnosis said:

It is not clear. I think it needs rewriting.

I'm not gonna lie, like when I was younger and like Sex-Ed and stuff, they didn't really like teach it. They taught me more like abstain. Because they're like the best way to prevent everything, just abstain.

4.3.4. Personal Background

Breaking the cycle of becoming a mother at a young age was especially important to participants whose mothers had experienced an unplanned pregnancy in their adolescence or early twenties. The hardship experienced by their mothers navigating young motherhood further exacerbated participants' fear of having an unplanned pregnancy. A twenty-year-old Hispanic college student shared how her mother's experience influenced her decision to be on a prescribed contraceptive.

My mom's experiences have also had a big impact on how breaking the cycle is actually important and how it's up to you whether you want to be better for yourself and for your family.

Similarly, a twenty-year-old Hispanic college student shared how her mother's openness about her own life experiences shaped her contraceptive decision-making.

…my mom is very open to her past life or her childhood, and she talks about how those experiences have like made her feel, and it just kind of like impacts me because…I don't want to like I guess disappoint her in the way where it's like I'm gonna do the same or like all her sacrifices are for nothing.

Relationships between participants and their mothers played a major role in how participants perceived STI risk. For example, participants who talked to their mothers about sex-related topics such as pregnancy and STI prevention methods were less likely to practice dual-

method contraceptive use and more likely to have had a previous STI diagnosis compared to participants who have openly discussed sex-related topics with their mother. Below, a nineteen-year-old college student with a prior STI diagnosis who identified as African-American, Hispanic, and White shared her experience of attempting to discuss sex and pregnancy prevention with her mother.

> *And my mom she just kind of was always kind of like, was kind of shushing me. She was like, oh no, like don't have sex like, I don't want to, I don't want to know about that, you have to be a virgin. Like she, she wanted, I think she would prefer if I was a virgin until I'm like 20, 23 or something.*

4.3.5. Making Assumptions: Participant Characteristics, Relationship Dynamic, and Defer to Partner

Instead of using condoms or inquiring about condom use, participants made assumptions about their partner's STI status based on specific partner characteristics. For example, age played a significant role in STI risk perception analysis and dual-method contraceptive decision-making as participants with older sexual partners were more likely to assume that their partner did not have an STI, resulting in less dual-method contraceptive use. Older partners were *mature* and *responsible*. However, they rarely used condoms or had conversations about condoms or STIs before engaging in sex with the participant. In the quote below, a twenty-two-year-old Hispanic college student recalled a conversation she had with a friend regarding her older sexual partner.

> *I had mentioned to her* [friend] *that I was seeing this older guy. He was 21 at the time. I was like, oh, I really liked him, like, we already have sex, but he tends to pull out. I was like, do you think that's okay?*

Additionally, participants with older partners were more likely to defer to their partners for condom use decision-making. Participants reported that their older partners had more sexual experience, contributing to an uneven power dynamic in the relationship. Below, a twenty-two-year-old Hispanic college student described how she feared being perceived negatively by her older partner if she inquired about condom use.

> *He was a little bit older than me, and I guess I was shy just because I was like, oh, he's more experienced. Let me not ask these questions: what if he looks down on me?*

Trustworthiness was another partner characteristic that participants factored into their STI risk perception analysis and dual-method contraceptive decision-making. For example, a twenty-year-old Hispanic college student explained how trust plays a vital role in her dual-method contraceptive use decision-making.

> *I personally don't really use condoms because the people who are my partners are usually people that I trust.*

Meanwhile, several participants attributed a prior STI diagnosis to a partner that they perceived as trustworthy. A nineteen-year-old college student who identified as African American, Hispanic, and White provided insight into a prior STI diagnosis:

> *The first time I ever had condomless sex, I trusted the person because they said they get tested regularly, and that was the first and last time I contracted an STI, which was chlamydia.*

Participants also made assumptions about their partner's STI status based on their partner's sexual history as participants were less likely to use condoms with partners whom they

believed to have had a low number of sexual partners. In the quote below, a twenty-year-old Hispanic college student assumed her partner's STI status based on his sexual history:

> *Because I feel like he hasn't been with many. At the top of my head probably like two to three before me, so it wasn't like, oh my god he's been with more than ten. I should probably have him get checked out before or anything like that.*

4.3.6. Relationship Dynamic

Monogamy, actual or perceived, was a major factor associated with dual-method contraceptive decision-making as participants reported less condom use while being in a perceived monogamous relationship compared to those who were having casual sex. Participants made assumptions about their partner's STI status based on their partners being perceived as *faithful* or *loyal.* Below, a twenty-year-old African American college student reflected on her thought process in a previous relationship.

> *I'm not gonna wear no condom with this person because he's probably never gonna cheat on me or leave me, you know?*

It is not clear. Needs rewriting.

Participants reported not wearing condoms while in non-monogamous sexual relationships or when having sex with a new partner, but not while in a monogamous relationship. For example, a twenty-year-old African American participant with a high school education said, *I just didn't use them* [condoms] *as much because I was in a relationship*.

In some instances, perceived monogamy played a role in participants' decision to defer to their partner for condom use decision-making. For example, an eighteen-year-old Hispanic participant with a high school education reported:

The sequesnce of the quotes doesn't guide the readr to think about the logic.

He started saying that we were already together, so what was the reason for us to, you know, use condoms if we were just with each other? But I mean, he did cheat on me, so, because obviously I got chlamydia.

Additionally, condom use was low in relationships where participants reportedly knew their partner for a period of time prior to engaging in sexual intercourse with them. In the quote below, a twenty-four-year-old African American college student provided insight into how familiarity played a role in her dual-method contraceptive decision-making.

Well, even before then, the person that gave me the last one [STI], *we used to date back in middle school. So, I felt comfortable with him, you know, it wasn't like some dude off the street.*

4.3.7. STI Testing as Reassurance

Frequent STI testing was common among participants who did not practice consistent condom use. Participants often assumed that STI testing (without condom use) was enough to protect them from STIs, Participants reported that STI testing provided them with *reassurance* after engaging in sexual intercourse without a condom. A twenty-four-year-old, Hispanic college graduate discussed her experience:

I would just go ahead and book an appointment literally for that week and tell the doctor what was going on, just because I'm very paranoid to some extent, like because I knew I was, like, having casual sex [without a condom].

Additionally, some participants decided to completely stop wearing condoms because they were getting tested frequently for STIs. In the quote below, a twenty-three-year-old, Hispanic high school graduate with a prior STI diagnosis explained further:

These two highlighted part has conflict and reject the other one.

Yeah, I'm way more comfortable not using condoms anymore. Yeah, but again, I'm emphasizing like I'm emphasizing getting a screen or getting tested with my partner or making sure that they've been tested recently and then if I do just have sex with them, I make sure I'm like, okay in three months, I'm gonna get tested and I'll see

Participants who continued to have sex with partners who had previously given them an STI preferred STI testing to using condoms. A twenty-four-year-old African American college student shared her experience:

Even when, like, when I found out my spouse was cheating, every time we had sex, I set a doctor's appointment, I don't care if it was every week, every two weeks, every three weeks, every month, I always set a doctor's appointment afterwards.

4.3.8. STI Diagnosis: A New Reality

For many participants, an STI diagnosis created a shift in STI risk perception analysis and dual-method contraceptive decision-making. As participants grappled with their STI diagnosis, they began to consider implementing behavioral changes to reduce their likelihood of acquiring another STI in the future. Below, a twenty-three-year-old Hispanic participant with a high school education explained:

And that kind of put me in the spot where it's kinda like, oh, I should start watching, you know, watching out who I mess with and who I, I should use more protection.

Similarly, a twenty-year-old Hispanic, college student shared:

So, I was like, in my mind, like, biological, thinking like I can't get pregnant on my period so I didn't really put too much effort into like using a condom, but after chlamydia, I'm like use a condom every time like it's not negotiable.

Following an STI diagnosis, some participants reported behavior change as they stopped deferring to their partners for condom use decision-making and started demanding condom use from their sexual partners. A twenty-two-year-old Hispanic college student shared her experience:

I kind of did put my foot down; I was like, look, it's either we're gonna wear condoms or we're just gonna leave it out here, I was like, I enjoy being with you, obviously, but if you are not gonna follow through with this, then there's no point of us being like that.

As participants began to take control of their health, they reported experiencing a newfound sense of confidence. For example, a twenty-year-old African American high school graduate said, *When you put your foot down, it feels empowering*. Similarly, a twenty-two-year-old Hispanic college student shared:

But you demanding a condom, it's like you know what you want in your life, you take care of yourself, you're aware of what can happen and consequences when having sex.

4.4. Discussion

This grounded theory study provides insight into the complex decision-making processes associated with STI risk perception analysis and dual-method contraceptive use among a racially and ethnically diverse group of AYACF. This study found that AYACF are making assumptions about the STI status of their sexual partners and deferring to them for condom use decision-making. Rather than inquire about their partner's STI status or ask them to wear a condom,

I don't remeber anything about lifelong commitment.

participants conducted their own risk perception analysis based on partner characteristics and relationship dynamics.

Specifically, perceived trustworthiness and monogamy were the most cited reasons for not practicing dual-method contraceptive use and deferring to partners for condom use decision-making. Further, among participants with a prior STI diagnosis, perceiving their partner as trustworthy and monogamous were the most common reasons for not using a condom during the sexual encounter that led to their STI diagnosis. A possible explanation for the relationship between perceived trustworthiness and monogamy and partner deferral is that young adult women are more likely to be attached to societal romantic love ideals such as love, faithfulness, and lifelong commitment in their relationship than their male partners, leading them to misjudge trust (52). Additionally, partner deferral is more likely to occur in relationships with an uneven power dynamic than in relationships where decision-making and power are equally distributed (53). Such findings were reflected in our study as participants who reported having older and more experienced sexual partners were more likely to defer to them for condom use decision-making.

An important finding in this study was the role mothers played in STI risk perception and dual-method contraceptive decision-making. More participants with mothers who avoided sexual health discussions were diagnosed with an STI than participants whose mothers engaged in conversations about sexual health-related topics such as STIs and pregnancy prevention. Further, more participants whose mothers personally experienced or had a close family member experience unintended pregnancy as an adolescent or young adult had discussed STI and pregnancy prevention with their daughters compared to mothers who had not experienced an unintended pregnancy at a young age. Mother-daughter communication reduces STI and

It is necessary to address HIV differently, as it is not a short term disease.

unintended pregnancy among adolescents under 18 years of age (54-56). Our findings demonstrate that mother-daughter communication in adolescence may also reduce STI and unintended pregnancy risk in young adulthood. Considering the powerful role mothers played in STI and pregnancy prevention, future research should focus on how to facilitate sexual health communication between mothers and daughters.

Another finding from this study is that participants used STI testing as reassurance after engaging in sexual intercourse without a condom. Conversely, a previous study found that young adult women were using STI testing as a form of reassurance before engaging in sexual intercourse without a condom (9). One possible explanation for participants using STI testing as reassurance is that participants were recruited from clinics with free sexual health services (including STI testing), eliminating any potential financial barriers to STI testing.

Another possible explanation for participants using STI testing for reassurance after engaging in sexual intercourse without a condom is that participants perceive STIs as temporary illnesses that are treatable or curable, as opposed to chronic. Further, frequent STI testing might be used as a substitue for engaging in condom negotiation with partners prior to sex, a task that could be viewed as uncomfortable or confrontational. Future research should focus on the role access to free STI testing plays in dual-method contraceptive decision-making and STI risk perception, and how patients are receiving STI education at clinics that offer free sexual health services.

Additionally, societal factors such as state abortion policy and school-based sex education may have played a significant role in perpetuating fear of unplanned pregnancy among participants. Our study found that recent changes to abortion laws likely exacerbated participants' pre-existing fear of pregnancy as participants reported buying pregnancy tests and Plan B in

Prior to the provision of free testing, people should be informed that it can't be an alternative for prevention.

The discussed sexual relation is an interaction between a male and female. Interview with some male was necessary.

bulk, as well as considering potentially life-threatening measures to self-induce an abortion after Roe V. Wade was overturned. Meanwhile, almost all participants reported having a sex education that focused on abstinence and avoiding pregnancy but did not provide information about STIs or STI prevention. Thus, their reduced fear of STIs in comparison to pregnancy might be attributed to a lack of knowledge about STIs and their health-related consequences.

4.4.1. Limitations

This study has limitations. First, the majority of participants were in college and identified as Black/African American and/or Hispanic, and therefore the findings may not be applicable to AYACF who do not attend college and/or belong to other racial or ethnic groups. Second, the majority of participants had access to free STI testing and treatment and participants might not have felt as much of a need to protect themselves from STIs as AYACF without access to free STI testing and treatment. Third, this study was cross sectional, limiting the understanding of the participants' behavior over a period of time.

4.4.2. Conclusion

Our results indicate that dual-method contraceptive decision-making and STI risk perception analysis is a complex process involving personal, relationship, and societal factors. AYACF likely have low STI risk perception, are not practicing dual-method contraceptive use, and are deferring condom use decision-making to partners they perceive as trustworthy or monogamous. Once diagnosed with an STI, AYACF likely stop relying on perceptions and assumptions to determine the need for dual-method contraceptive use and begin to engage in new thinking and behaviors. The shift in perspective and behavior caused by the STI diagnosis

Working on the false rumor is important too.

highlights the need to empower young women to put their own sexual health needs before their partners.

Additionally, STI education is important in reducing STI risk. Mother-daughter communication may increase dual-method contraceptive use, reducing STI risk. Future research should focus on ways to promote mother-daughter communication about STI prevention in the AYACF population.

References

1. DSHS TDoSHS. Texas STD Surveillance Report: 2022 Annual Report. Texas Department of State Health Services; 2024.

2. CDC CfDCaP. Teen Birth Rate by State 2022 [Available from: https://www.cdc.gov/nchs/pressroom/sosmap/teen-births/teenbirths.htm.

3. Fuentes L, Ingerick M, Jones R, Lindberg L. Adolescents' and young adults' reports of barriers to confidential health care and receipt of contraceptive services. Journal of Adolescent Health. 2018;62(1):36-43.

4. CDC CfDCaP. Contraception 2020 [Available from: https://www.cdc.gov/reproductivehealth/contraception/index.htm.

5. CDC CfDCaP. Diagnoses of HIV Infection in the United States and Dependent Areas, 2021: Table 7B. Diagnoses of HIV infection attributed to heterosexual contact by year of diagnosis, assigned sex at birth, race/ethnicity and selected characteristics, 2017-2021-United STates and 6 dependent areas: Centers for Disease Control and Prevention; 2023 [Available from: https://www.cdc.gov/hiv/library/reports/hiv-surveillance/vol-34/content/tables.html.

6. CDC CfDCaP. STDs and HIV – CDC Fact Sheet 2021 [Available from: https://www.cdc.gov/std/hiv/stdfact-std-hiv-detailed.htm.

7. Copen CE. Condom Use During Sexual Intercourse Among Women and Men Aged 15-44 in the United States: 2011-2015 National Survey of Family Growth. Natl Health Stat Report. 2017(105):1-18.

8. Martinez GM AJ. Sexual activity and contraceptive use among teenagers aged 15–19 in the United States, 2015–2017. National Center for Health Statistics; 2020.

9. Lemoine J, Teal SB, Peters M, Guiahi M. Motivating factors for dual-method contraceptive use among adolescents and young women: a qualitative investigation. Contraception. 2017;96(5):352-6.

10. Cates WJ, Steiner MJ. Dual Protection Against Unintended Pregnancy and Sexually Transmitted Infections: What Is the Best Contraceptive Approach? Sexually Transmitted Diseases. 2002;29(3):168-74.

11. Walsh-Buhi ER, Helmy HL. Trends in long-acting reversible contraceptive (LARC) use, LARC use predictors, and dual-method use among a national sample of college women. Journal of American College Health. 2018;66(4):225-36.

12. Higgins JA, Cooper AD. Dual use of condoms and contraceptives in the USA. Sexual Health. 2012;9(1):73-80.

13. Pazol K, Daniels K, Romero L, Warner L, Barfield W. Trends in Long-Acting Reversible Contraception Use in Adolescents and Young Adults: New Estimates Accounting for Sexual Experience. J Adolesc Health. 2016;59(4):438-42.

14. Garnett C, Pollack L, Rodriguez F, Renteria R, Puffer M, Tebb KP. The Association Between Nonbarrier Contraceptive Use and Condom Use Among Sexually Active Latina Adolescents. Journal of Adolescent Health. 2021;68(5):985-90.

15. Pazol K, Kramer MR, Hogue CJ. Condoms for Dual Protection: Patterns of Use with Highly Effective Contraceptive Methods. Public Health Reports. 2010;125(2):208-17.

16. Sieving RE, Bearinger LH, Resnick MD, Pettingell S, Skay C. Adolescent Dual Method Use: Relevant Attitudes, Normative Beliefs and Self-Efficacy. Journal of Adolescent Health. 2007;40(3):275.e15-.e22.

17. Steiner RJ, Liddon N, Swartzendruber AL, Rasberry CN, Sales JM. Long-Acting Reversible Contraception and Condom Use Among Female US High School Students: Implications for Sexually Transmitted Infection Prevention. JAMA Pediatrics. 2016;170(5):428-34.

18. McNicholas CP, Klugman JB, Zhao Q, Peipert JF. Condom use and incident sexually transmitted infection after initiation of long-acting reversible contraception. American Journal of Obstetrics and Gynecology. 2017;217(6):672.e1-.e6.

19. Williams DJ, Noyes JM. How does our perception of risk influence decision-making? Implications for the design of risk information. Theoretical Issues in Ergonomics Science. 2007;8(1):1-35.

20. Hickey MT, Cleland C. Sexually transmitted infection risk perception among female college students. Journal of the American Association of Nurse Practitioners. 2013;25(7):377-84.

21. Schaefer R, Thomas R, Maswera R, Kadzura N, Nyamukapa C, Gregson S. Relationships between changes in HIV risk perception and condom use in East Zimbabwe 2003–2013: population-based longitudinal analyses. BMC Public Health. 2020;20(1):756.

22. Mehrotra P, Noar SM, Zimmerman RS, Palmgreen P. Demographic and personality factors as predictors of HIV/STD partner-specific risk perceptions: implications for interventions. AIDS Educ Prev. 2009;21(1):39-54.

23. Committee on Practice Bulletins-Gynecology L-ARCWG. Practice Bulletin No. 186: Long-Acting Reversible Contraception: Implants and Intrauterine Devices. Obstet Gynecol. 2017;130(5):e251-e69.

24. Menon S, ADOLESCENCE CO, Alderman EM, Chung RJ, Grubb LK, Lee J, et al. Long-Acting Reversible Contraception: Specific Issues for Adolescents. Pediatrics. 2020;146(2).

25. CDC CfDCaP. Sexually Transmitted Disease Surveillance 2019. Atlanta Centers for Disease Control and Prevention; 2021.

26. WHO WHO. Social Determinants of Health: World Health Organization; 2024 [Available from: https://www.who.int/health-topics/social-determinants-of-health#tab=tab_1.

27. WHO WHO. A Conceptual Framework for Action on the Social Determinants of Health 2010 [Available from: https://www.who.int/sdhconference/resources/ConceptualframeworkforactiononSDH_eng.pdf.

28. Lindberg LD, Kantor LM. Adolescents' Receipt of Sex Education in a Nationally Representative Sample, 2011–2019. Journal of Adolescent Health. 2021.

29. Crooks N, Wise A, Frazier T. Addressing sexually transmitted infections in the sociocultural context of black heterosexual relationships in the United States. Social Science & Medicine. 2020;263:113303.

30. Pequegnat W, Stover E. Considering women's contextual and cultural issues in HIV/STD prevention research. Cultural Diversity and Ethnic Minority Psychology. 1999;5(3):287-91.

31. Ellison JE, Brown-Podgorski BL, Morgan JR. Changes in Permanent Contraception Procedures Among Young Adults Following the Dobbs Decision. JAMA Health Forum. 2024;5(4):e240424-e.

32. Mitchell JA, Yao M, Maeda R, Lappen JR, Brant AR. Permanent and long-acting reversible contraception volumes at a multihospital system in Ohio before and after Dobbs. Contraception. 2024:110471.

33. Qato DM, Myerson R, Shooshtari A, Guadamuz JS, Alexander GC. Use of Oral and Emergency Contraceptives After the US Supreme Court's Dobbs Decision. JAMA Network Open. 2024;7(6):e2418620-e.

34. Guttmacher. Sex and HIV Education 2021 [Available from: https://www.guttmacher.org/state-policy/explore/sex-and-hiv-education.

35. Gavey N, McPhillips K, Doherty M. "If it's not on, it's not on"—or is it? Discursive constraints on women's condom use. Gender & Society. 2001;15(6):917-34.

36. Jarama SL, Belgrave FZ, Bradford JB, Young M, Honnold JA. Family, cultural and gender role aspects in the context of HIV risk among African American women of unidentified HIV status: An exploratory qualitative study. AIDS Care. 2007;19:307 - 17.

37. Fantasia HC. Influences of Social Norms and Context on Sexual Decision Making Among Adolescent Women. Journal of Midwifery & Women's Health. 2011;56(1):48-53.

38. East L, Jackson D, O'Brien L, Peters K. Condom negotiation: experiences of sexually active young women. J Adv Nurs. 2011;67(1):77-85.

39. Tyler CP, Whiteman MK, Kraft JM, Zapata LB, Hillis SD, Curtis KM, et al. Dual Use of Condoms With Other Contraceptive Methods Among Adolescents and Young Women in the United States. Journal of Adolescent Health. 2014;54(2):169-75.

40. Eisenberg DL, Allsworth JE, Zhao Q, Peipert JF. Correlates of Dual-Method Contraceptive Use: An Analysis of the National Survey of Family Growth (2006–2008). Infectious Diseases in Obstetrics and Gynecology. 2012;2012:717163.

41. Wilson EK, Koo HP. Associations Between Low-Income Women's Relationship Characteristics and Their Contraceptive Use. Perspectives on Sexual and Reproductive Health. 2008;40(3):171-9.

42. Manlove J, Welti K, Karpilow Q. Relationship Violence Typologies and Condom Use in Young Adult Dating Relationships. Perspectives on Sexual and Reproductive Health. 2019;51(2):109-17.

43. Kusunoki Y, Barber JS, Gatny HH, Melendez R. Physical Intimate Partner Violence and Contraceptive Behaviors Among Young Women. J Womens Health (Larchmt). 2018;27(8):1016-25.

44. Charmaz K. Constructing grounded theory: sage; 2014.

45. Bureau USC. QuickFacts: Harris County. 2021.

46. Medicine BCo. Teen Health Clinic. 2021.

47. TDHHS TDoHaHS. Sexually Transmitted Disease in Texas. 2020.

48. Cohen, Crabtree. Qualitative Research Guidelines Project 2006 [Available from: http://www.qualres.org/HomeSemi-3629.html.

49. Bolton M, McKay A, Schneider M. Relational influences on condom use discontinuation: A qualitative study of young adult women in dating relationships. The Canadian Journal of Human Sexuality. 2010;19(3):91.

50. Lumivero. NVivo 12. 2017.

51. Chun Tie Y, Birks M, Francis K. Grounded theory research: A design framework for novice researchers. SAGE Open Med. 2019;7:2050312118822927-.

52. Meier A, Hull KE, Ortyl TA. Young Adult Relationship Values at the Intersection of Gender and Sexuality. Journal of Marriage and Family. 2009;71(3):510-25.

53. Woolf SE, Maisto SA. Gender Differences in Condom Use Behavior? The Role of Power and Partner-Type. Sex Roles. 2008;58:689-701.

54. Teitelman AM, Calhoun J, Duncan R, Washio Y, McDougal R. Young women's views on testing for sexually transmitted infections and HIV as a risk reduction strategy in mutual and choice-restricted relationships. Appl Nurs Res. 2015;28(3):215-21.

55. Teitelman AM, Ratcliffe SJ, Cederbaum JA. Parent—Adolescent Communication About Sexual Pressure, Maternal Norms About Relationship Power, and STI/HIV Protective Behaviors of Minority Urban Girls. Journal of the American Psychiatric Nurses Association. 2008;14(1):50-60.

56. Hutchinson M. The Influence of Sexual Risk Communication Between Parents and Daughters on Sexual Risk Behaviors*. Family Relations. 2004;51:238-47.

Figure 1

Click here to access/download;Figure;Figure 1.docx

Figure 1. Dual-method Contraception Decision-Making Factors

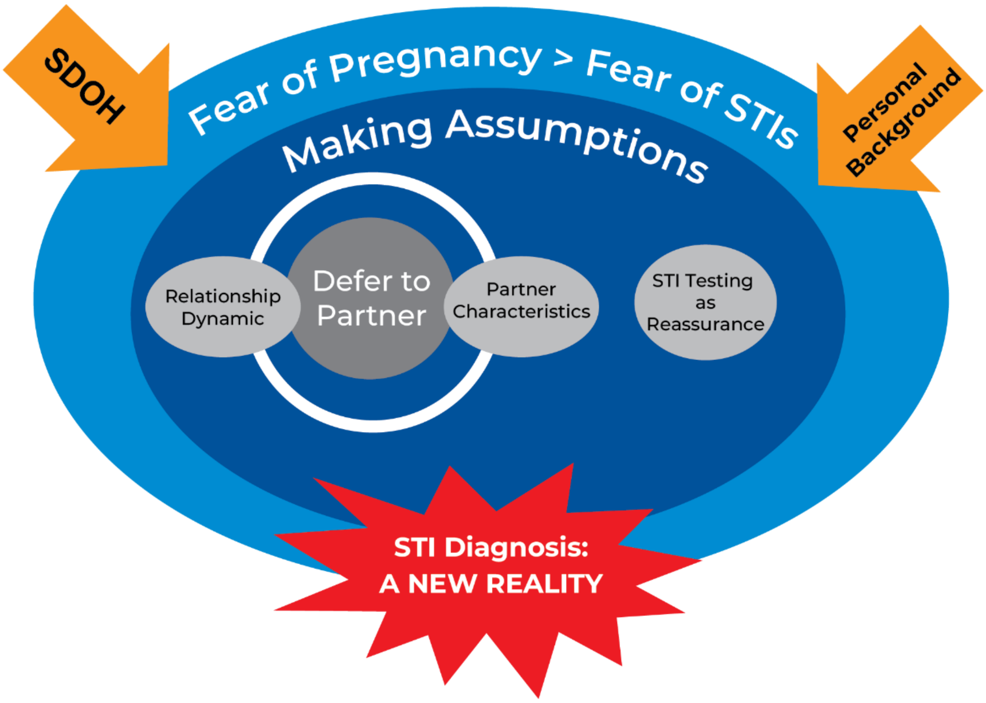

Ethics Approval

Click here to access/download
Supporting Information
Outcome_Letter.pdf

Revised Manuscript with Track Changes

The Theoretical Understanding of Risk Perception and Dual-Method Contraceptive Decision-Making among Texas Adolescent and Young Adult Cisgender Females

Formatted

Lauren Holt, PhD, MSN, RN[a], Amy Corneli, PhD, MPH[b], Eleanor Stevenson, PhD, RN, FAAN[a], Jonas J. Swartz, MD, MPH[c], and Michael Relf, PhD, RN, ACNS-BC, CNE, ANEF, FAAN[a]

[a] School of Nursing, Duke University School of Nursing, 307 Trent Drive, Durham, NC 27710; lauren.holt@duke.edu, eleanor.stevenson@duke.edu, michael.relf@duke.edu
[b] Department of Population Health Sciences, 215 Morris Street, Suite 210, Durham, NC 27701; amy.corneli@duke.edu
[c] Department of Obstetrics & Gynecology, Duke University Medical Center, 201 Trent Drive, 203 Baker House, Durham, NC 27710; jonas.swartz@duke.edu

Corresponding author:
Lauren Holt, PHD, RN
3401 Webberville Rd.
Austin, TX 78702
P 512-223-57831310 Karen Ave.

Austin, TX 78757
P 325.650.8282
Lauren.holt427@gmail.comduke.edu

Formatted: Right: 0.25"

Abstract

In this paper we use a constructivist grounded theory approach to conduct individual, qualitative interviews with a racially and ethnically diverse group of adolescent and young adult cisgender females (AYACF) (18 to 24 years of age) to generate theoretical knowledge of the relationship between Social Determinants of Health (SDOH), STI and HIV risk perception, and dual-method contraceptive decision-making (the use of, both, a prescribed contraceptive [commonly referred to as "birth control"] and a condom during vaginal intercourse). Twenty-five AYACF were selected through theoretical sampling. Data were collected using semi-structured interviews and analyzed using constant comparison. AYACF expressed having a greater fear of pregnancy than sexually transmitted infections (STIs). They described deferring to their partners and making assumptions about their partner's STI status based on partner and relationship factors, therefore not needing to engage in condom negotiation.Mother-daughter sexual health conversations and state school-based sex education and abortion policies influenced participants' STI risk perception and dual-method contraceptive decision-making. Participants who were diagnosed with an STI reported experiencing a shift in perception about their likelihood of acquiring an STI, often resulting in behavior change. These findings are important as they provide insight into the complex decision-making processes, influenced by SDOH, that a racially and ethnically diverse group of AYACF associate with STI risk perception and dual-method contraceptive use.

In this paper we use describe a constructivist grounded theory approach to examine the factors adolescent and young adult cisgender females (AYACF) (18 to 24 years of age) consider when

Formatted: Font: 12 pt, Not Bold

Formatted: Font: 12 pt, Not Bold

Formatted: Font: 12 pt, Not Bold

Formatted: Right: 0.25"

deciding to engage in dual-method contraceptive use (the use of, both, a prescribed contraceptive [(commonly referred to as "birth control"]) and a condom during vaginal intercourse). Tselected through . Data were collected using analyzed using Our findings suggest that AYACF expressed havinge a greater fear of pregnancy than sexually transmitted infections (STIs). They described and are making assumptions about their partner's STI status based on partner and relationship factors therefore rather than not needing to engage in condom negotiation. Additionally, Social Determinants of Health such as mMother-daughter sexual health conversations and state school-based sex education and abortion policies influenced participants' STI risk perception and dual-method contraceptive decision-making. Participants who were diagnosed with an STI reported experiencing a shift in perception about their likelihood of acquiring an STI, sometimes resulting in behavior change. Such These findings are significant important as they provide insight into the into the complex decision-making processes, influenced by social determinants of health, that a racially and ethnically diverse group of AYACF associated with STI risk perception analysis and dual-method contraceptive use among a racially and ethnically diverse group of AYACF.

~~1.~~ Introduction

Sexually transmitted infections (STIs), including HIV, are a significant public health concern. In Texas, STIs are on the rise as primary syphilis rates doubled over the last five years, and congenital syphilis rates are at an all-time high (1). Adolescent and young adult cisgender females (AYACF) between 18 and 24 years old have experienced a consistent increase in syphilis over the last five years, while accounting for higher rates of chlamydia than any age or gender group (1). Further, AYACF belonging to structurally oppressed racial and ethnic groups are disproportionately more affected by STIs and HIV than White AYACF; ~~, as~~ Black females are five times more likely to be diagnosed with chlamydia and three times more likely to be diagnosed with HIV, while Hispanic females are twice as likely to be diagnosed with syphilis (2). Moreover, American Indian/Alaskan Native females are six times more likely to be diagnosed with gonorrhea, and five times more likely to be diagnosed with syphilis than their White counterparts (2).

Despite rising STI rates, pregnancy rates have steadily declined among Texas AYACF (3), ~~–~~a feat largely attributed to an increase in access and utilization of prescribed contraceptive methods ~~methods~~ (i.e intrauterine devices (IUDs), birth control pills, hormonal implants, injections, patches, and rings) (3-5). Although prescribed contraceptives are 90-99% effective effective in reducing the risk of pregnancy when used correctly (6), they do not protect against STIs or HIV.

The risk of acquiring an STI and HIV can be reduced through correct and consistent condom use during oral, vaginal, and anal sex (7). However, only 23% of AYACF report using~~e~~ condoms during every sexual encounter compared to those who use condoms consistently ~~-~~(8).

Formatted: Font: 18 pt, Bold

Formatted: Indent: First line: 0.3", Space After: 3 pt, Don't add space between paragraphs of the same style

Formatted: Right: 0.25"

Further, AYACF who use prescribed contraceptives are less likely to practice dual-method contraceptive use (9, 10), which occurs when a prescribed contraceptive and a condom are both used during intercourse (11), and . Further, adolescent females who use long-acting reversible contraceptives (LARCs) such as IUDs and implants are less likely to practice dual-method contraceptive use and have more sexual partners than those using short-acting reversible contraceptives (SARCs) such as oral birth control pills (12-15) (16).

Increasing trends in STI rates and limited dual-method contraceptive use highlight the need to investigate attitudes and beliefs related to contraceptive use and STI and HIV risk perception among AYACF. Risk perception analysis is an important factor in dual-method contraceptive decision-making as AYACF with a high STI and HIV risk perception are more likely to initiate dual-method contraceptive use than those who perceive themselves as low risk (13, 17-19). While risk perception analysis has been identified as an integral part of decision-making (20), the processes AYACF associated with STI and HIV risk perception analysis and dual-method contraceptive decision-making among AYACF are poorly understood.

The overall objective of this qualitative study was to examine the factors adolescent and young adult cisgender females (AYACF) (18 to 24 years of age) consider when deciding to engage in dual-method contraceptive use. Using a constructivist grounded theory approach, we conducted individual, qualitative semi-structured interviews with a racialy and ethnically diverse group of sexually active AYACF using prescribed contraceptives to generate theoretical knowledge of the relationship between Social Determinants of Health (SDOH), STI and HIV risk perception, and dual-method contraceptive decision-making to inform the development of a conceptual model for future theoretical testing and intervention development.

Formatted: Right: 0.25"

Dual-method contraceptive use is more likely to occur in women with a high STI and HIV risk perception than those who perceive themselves as low risk (17). Further, women with low STI and HIV risk perception are more likely to engage in high-risk sexual behaviors (13, 18, 19). Previous studies have shown risk perception to be a determinant factor for engaging in STI and HIV protective behaviors (21-23). Risk perception plays an integral role in the decision-making process (20).However, the processes associated with risk perception and dual-method contraceptive decision-making are poorly understood. Moreover, as professional associations and practice guidelines continue to promote LARCs as the first-line contraceptive method among adolescent women (24, 25), more research is needed to understand how the utilization of prescribed contraceptives impacts one's perceived risk of acquiring an STI and the processes associated with dual-method contraceptive use decision making. constructivist semi-structured

Formatted: Indent: First line: 0.5", Space After: 8 pt

4.1.1. Background: Social Determinants of HealthSTI and HIV Disparities

Formatted: Font: 16 pt, Bold

AYACF belonging to structurally oppressed racial and ethnic groups are disproportionately more affected by STIs than White AYACF. For example, Black females are five times more likely to be diagnosed with chlamydia and three times more likely to be diagnosed with HIV when compared to White females, while Hispanic females are twice as likely to be diagnosed with syphilis (2). Moreover, American Indian/Alaskan Native females are six times more likely to be diagnosed with gonorrhea, and five times more likely to be diagnosed with syphilis than their White counterparts (2). The existing age, gender, racial, and ethnic STI and HIV disparities highlight the need to consider how social determinants influence dual-method contraceptive use among AYACF.

Formatted: Right: 0.25"

The World Health Organization (WHO) defines ~~the~~ SDOH as the circumstances under which individuals are born, raised, live, work, and age, and the available systems to deal with illness (21). These circumstances are shaped by structural determinants which encompass social and political mechanisms that influence individual and collective access to resources, further contributing to social class division (22). Structural determinants such as statewide policies pertaining to sexual and reproductive health access and education, and societal and cultural norms and values influence dual-method contraceptive use among AYACF, resulting in STI and HIV disparities (23-25). For example, only 18 states require sex education content to be medically accurate and only 27 states require HIV education to be included in sex education curricula (26). Further, only 20 states are required to provide students with information on contraceptive methods (26). As a result, only 52% of U.S. youth receive education about where to obtain prescribed contraceptive methods and only 59% are educated on condom application (23).

Additionally, abortion policy has influenced contraceptive decision-making, as the ~~overturning~~overturn of *Roe V. Wade* in June 2022 ~~has lead cisgender females and people with uteruses (and cisgender men) to re-evaluate their pregnancy prevention methods~~prompted re-evaluation of pregnancy prevention for many. For example, since 2022, LARC use has increased, and the number of tubal sterilization and vasectomy procedures increased among young adults ages 18 to 30 years old (27, 28). Moreover, the use of emergency contraception (commonly referred to as Plan B) surged after June 2022 (and has since subsided) while oral contraceptives have decreased in states where abortion laws are the most restrictive (29).

Formatted: Indent: First line: 0.3"

Formatted: Indent: First line: 0.3"

Formatted: Font: Italic

Formatted: Right: 0.25"

~~Since Roe V. Wade was overturned in June 2022, abortion because a state's right issue, and thus became illegal in twelve states, including Texas, causing cisgender women and people with uteruses (and cisgender men) to re-evaluate their pregnancy prevention methods. For example, over the last two years LARC use has increased, and the number of tubal sterilization and vasectomy procedures increased among young adults ages 18 to 30 years old (31, 32). Additionally, the use of emergency contraception (commonly referred to as Plan B) surged after June 2022 (and has since subsided) while oral contraceptives have decreased in states where abortion laws are the most restrictive (33).~~

~~Sex education also plays a major role in dual-method contraceptive decision-making. Currently, only 18 states require sex education content to be medically accurate and only 27 states require HIV education to be included in sex education curricula (31). Further, only 20 states are required to provide students with information on contraceptive methods (31). As a result, only 52% of U.S. youth receive education about where to obtain prescribed contraceptive methods and only 59% are educated on condom application (28). While there is a strong correlation between inadequate sexual health education policy and climbing STI and HIV rates, societal and cultural norms and values also play an integral role in determining STI and HIV risk and disparities.~~

Societal and cultural norms and values are structural determinants which determine STI and HIV risk and can inadvertently serve as a barrier to engaging in dual-method contraceptive use, if not acknowledged (24, 30, 31). Gendered societal expectations (also called "gender roles") consist of how society expects women and men to behave, express themselves, and interact with others (32). For example, in most cultures women are expected and perceived to be selfless, polite, and submissive, while men are expected to be strong, assertive, and dominant (32). As a

Formatted: Right: 0.25"

result of gendered societal expectations, uneven relationship dynamics and are likely to occur, resulting in women feeling less empowered to negotiate condom use ~~Gendered societal expectations and sexual stereotypes are the foundation of uneven relationship dynamics, resulting in the decreased use of condoms and/or prescribed contraceptives~~ (24, 25, 33, 34). ~~Further, gendered societal expectations and sexual stereotypes are disproportionately experienced by AYACF belonging to structurally oppressed racial and ethnic groups, contributing to glaring STI and HIV disparities (29).(40, 41)(41)~~

In the context of SDOH, structural determinants operate through intermediary determinants which directly affect dual-method contraceptive use among AYACF (22). Health care access and quality play a critical role in determining dual-method contraceptive use as AYACF with access to health insurance are more likely to engage in dual-method contraceptive use and less likely to report an STI and HIV than those who are medically uninsured (35, 36).

Additionally, psychosocial factors such as the interpersonal relationship dynamics between an AYACF and her partner determine dual-method contraceptive use as AYACF who report higher levels of relationship trust and commitment are less likely to practice dual-method contraceptive use than those in less trusting or committed relationships (13, 37). Meanwhile, negative relationship factors such as violence and conflict are associated with inconsistent, or lack of, dual-method contraceptive use (38, 39). To understand the processes ~~associated~~ influencing with dual-method contraceptive decision-making and STI and HIV risk perception among AYACF, healthcare access, and quality and relationship factors must be taken into consideration.

~~4.1.2. Study Purpose~~

Formatted: Font: 18 pt, Bold

Formatted: Right: 0.25"

The overall objective of this qualitative study was to gain insight into STI and HIV- related health factors among a racially and ethnically diverse AYACF population who are using prescribed contraceptives. Using a grounded theory approach, we conducted individual qualitative interviews with sexually active AYACF using prescribed contraceptives to generate theoretical knowledge of the relationship between SDOH, STI and HIV risk perception, and decision-making and use that knoweldge to inform the development of a conceptual model for future theoretical testing and intervention development. Here we report on the following two aims of the larger study:

Aim 1: Understand AYACF's STI and HIV risk perception and decision-making related to dual-method contraceptive use.

Formatted: Right: 0.25"

~~Aim 2: Develop a conceptual model explaining the processes associated with risk perception analysis and decision-making related to dual-method contraceptive use among sexually active AYACF using prescribed contraceptives.~~

~~4.2.~~ Methods

~~4.2.1.~~ Study Design

This study implemented a constructivist grounded theory qualitative design to generate a middle-range theory related to STI and HIV risk perception analysis and dual-method contraception decision-making among sexually active AYACF (40). ~~Following constructivist grounded theory procedures, T~~the proposed study integrated the essential components by 1) theoretically sampling participants to ~~get representativeness of the population~~further explore emerging data, 2) conducting in-depth ~~intensive~~ interviews, 3) developing and using memos to ensure dependability and confirmability in increasing the trustworthiness of the data and findings

Formatted: Font: 18 pt, Bold

Formatted: Font: 16 pt, Bold

Formatted: Right: 0.25"

and, 4) using a constant comparison approach in data analysis (40). The primary data collection strategy was~~ intensive~~ individual semi-structured qualitative interviews collected at one point in time to provide an in-depth exploration of the participants' experiences and situations (40).

Unlike Classic or Straussean grounded theory approaches, constructivist grounded theory acknowledges that the researcher is not neutral in their observations or a value-free expert, requiring that the researcher address how their own privileges and preconceptions may shape the data analyses (40). A constructivist approach was most suitable for this study as the author's own experience as an AYACF with prior academic knowledge of prescribed contraceptive use needed to be taken into consideration throughout data collection and analysis.

~~4.2.2.~~ Participants and Setting

Formatted: Font: 16 pt, Bold

In conducting a constructivist grounded theory study, it was imperative to select participants who had first-hand experience that would address the aims of the proposed study (40). To understand the processes of STI and HIV risk perception and dual-method contraceptive decision-making among sexually active AYACF using prescribed contraceptives, participants must ~~had~~ have ~~to~~ m~~e~~et the following inclusion criteria: 1) self-identify as a cisgender female; 2) be 18 to 24 years old; 3) report vaginal intercourse in the last 12 months; and 4) currently using a prescribed contraceptive method such as LARCs (i.e. IUD's, implants) or SARCs (i.e. contraceptive pills, injections, patches, rings).

Study recruitment occurred from January 11, 2023 to November 30, 2023 ~~11/01/2023 to 30/11/2023~~ at a health clinic in urban Texas that provides free gynecological, birth control, family planning, and STI screening and treatment services to adolescents and young adults in one of the largest and most racially and ethnically diverse counties in the US (41, 42). Further,

Formatted: Right: 0.25"

the county where the health clinic resides reports the highest STI case numbers in the state of Texas while accounting for 1 in 4 new HIV diagnoses at a rate that is almost twice the state average (43).

Descritpion of ~~4.2.3.~~ Data Collected~~ion~~

A semi-structured interview format was appropriate for this study as it facilitated the exploration of researcher-initiated topics~~data collection~~, while still allowing participants to freely discuss thoughts or ideas that might not have been considered by the research team (44). Interview questions were drafted by the research team who specialize in sexual and reproductive health to ensure content validity. Further, participant input contributed to the development of additional questions that were asked in future interviews. After the fourteenth interview, triangulation was used to identify new questions to add to the interview guide that would elicit further discussion about perceived monogamy and STI risk. While searching for qualitative studies that focused on condom use among adolescents and young adults, the research team identified a study by Bolton, McKay (45) and adapted questions from their interview guide starting with the fifteenth interview. Based on observations from the first fourteen interviews, the research team added questions such as “How did you know you were/are in a monogamous relationship?”,” What was happening in the relationship at the time?” and “What do you think is the likelihood that you will acquire an STI?” (45). Key questions that guided the interviews are presented in **Table 1**. The addition of these questions provided further insight into how the participants perceived their partners and themselves while in a romantic and/or sexual relationship, guiding their contraceptive decision-making.

Formatted: Font: 16 pt, Bold

Formatted: Font: 16 pt, Bold

Formatted: Indent: First line: 0.5", Line spacing: Double

Formatted: Right: 0.25"

Table. 1. Social Determinants of Health Interview Guide

Rapport-building questions:<br>• Tell me a little about yourself.<br>• What interested you in this study?
Structural Determinants<br><br>STI/HIV Prevention *(Sex Education Policy/Societal and Cultural Norms and Values)*<br>Question 1: As previously mentioned in the screening, this is a study for sexually active women who are using prescribed contraceptives, commonly referred to as birth control, and we are interested in learning how they perceive STI/HIV risk. What do you think are the most important ways a young woman can protect herself against pregnancy and sexually transmitted infections (STIs) including HIV?<br>• Probe: Tell me about your experience in learning about prescribed contraceptive and condom use.<br>• Probe: Tell me about your sexual health education experience in school<br>• Probe: Can you share with me how women in your community protect themselves from pregnancy and STIs, including HIV.<br>• *Probe: Tell me about how one's culture might influence using a birth control and/or condom during sex*<br>• Probe: How might where someone grew up influence their understanding of sexual health and prescribed birth control
Intermediary Determinants<br><br>Prescribed Contraceptives *(Health System and Psychosocial)*<br>Question 2: Could you tell me about the events that led to your decision to seek prescribed contraceptive?<br>• Probe: As you thought about seeking prescribed contraceptives, who were the individuals you talked to for guidance?<br>• Probe: Can you share with me the information you were considering before starting a prescribed contraceptive?<br>• Probe: Tell me about any barriers you faced in obtaining your prescribed contraceptive?<br>• Probe: Tell me about your interaction with your provider during the visit you were prescribed contraceptive<br>• Probe: Tell me about the things you were thinking about when you made the decision to start a specific prescribed contraceptive.<br><br>Prescribed Contraceptive and Condom Use *(Psychosocial)*<br>Question 3: How would you describe how you viewed condom use before you got on a prescribed contraceptive?

Formatted: Right: 0.25"

- Probe: What pregnancy prevention methods did you use before starting a prescribed contraceptive?
- Probe: What contributed to your decision to use (or not to use) condoms before starting a prescribed contraceptive?
- Probe: Could you tell me about how your views towards condom use may have changed since starting a prescribed contraceptive?

Sexual Partners *(Psychosocial)*

Question 4: Since we have been discussing condom use, I would like to learn about your current or most recent sexual partner.

- Probe: Can you share with me conversations that you and your partner had regarding sexually transmitted infection (STI) prevention?
- Probe: Can you tell me how you both came to this understanding?

For participants who are currently in or had previously been in a monogamous relationship:

- *How did you know you were/are in a monogamous relationship?(45)
- *Probe: What was happening in the relationship at that time?(45)

For all participants:

- *What do you think is the likelihood that you will acquire an STI?(45)

Closing: Is there anything else you would like to share with me that we haven't talked about today?

- Before we end our conversation, I wanted to give you an opportunity to ask any questions.

*Questions inserted after fourteenth interview

Before the interviews~~s~~, each participant completed a demographic data form which included age, sex, gender identity, race and ethnicity information, relationship status, and type of prescribed contraception in use. ~~Individual semi-structured interviews took place virtually via Zoom and were recorded with an encrypted audio recording device. All interviews were transcribed verbatim by a transcription service and checked against the recording for accuracy. Investigator memos were written immediately following the interviews and supplemented throughout the data analysis process to capture thoughts and develop connections (40). For participating in the study, each participant was compensated with a $40 Amazon gift card.~~

Formatted: Right: 0.25"

~~4.2.4.~~ Study Procedures

Formatted: Font: 16 pt, Bold

Theoretical sampling was implemented based on age, race, ethnicity, and type of prescribed contraceptive (SARC vs. LARC) (40). A total of twenty-five participants were interviewed for this study. Fifteen of the ~~ll~~ selected participants were recruited in person at the health clinic and ten participants were ~~or~~ referred to the research team by other participants. Once participants agreed to the interview, they were provided with a consent form to be signed before the interview. Participants were given the option to be interviewed in a private room at the clinic in-person or virtually in a private location of their choice. A total of twenty-five one-time, semi-structured interviews were conducted via Zoom after obtaining informed consent. All twenty-five interviews were conducted by one member of the research team (LH) who has training in qualitative research methods. Following theoretical sampling procedures (40), after one interview was completed, transcribed, and analyzed, the next participant was theoretically recruited and selected for an interview to ensure the representativeness of cisgender adolescent young adult women's experiences. Characteristics of previous participants (i.e. age, race, ethnicity, education, birth control method) were components considered in the theoretical sampling process. This sampling approach helped to ensure the confirmability of diverse experiences.

Individual semi-structured interviews took place virtually via Zoom and were recorded with an encrypted audio recording device. All interviews were transcribed verbatim by a transcription service and checked against the recording for accuracy. Investigator memos were written immediately following the interviews and supplemented throughout the data analysis process to capture thoughts and develop connections (40). For participating in the study, each participant was compensated with a $40 Amazon gift card.

Formatted: Right: 0.25"

4.2.5. Data Analysis

An inductive qualitative content analysis, using constant comparison, was conducted to explain the processes AYACF consider associated with risk perception analysis and decision-making related to dual-method contraceptive use among sexually active AYACF using prescribed contraceptives (40). The transcribed verbatim transcripts were imported into NVivo for coding and analysis (46). Constant comparison analysis was performed between two members of the research team who have experience in qualitative data analysis and sexual and reproductive health research throughout the length of the data collection period and during three phases of coding- initial, focused, and theoretical.

First, iInitial coding was performed to ensure fit and relevance of the analyzed data and to identify connections between the participant's actions and larger social processes to help to identify the possible paths that the data might take (40). The initial coding process began shortly after an interview took place and consisted of line-by-line coding of the transcribed interviews, field notes, and memos in order to highlight recurring words and immediately compare and contrast findings from previous interviews (47). To add precision and clarity to the data analysis, the research team conducted theoretical coding by 1) analyzing and synthesizing relationships between focused codes and 2) building a conceptual model explaining the processes associated with risk perception analysis and decision-making related to dual-method contraceptive use among sexually active AYACF using prescribed contraceptives (44, 52). The development of the conceptual model began during the theoretical coding phase of the first interview and occurred throughout the theoretical coding phase of the remaining interviews. Next, the focused coding process occurred by assessing and comparing the initial codes to distinguish which codes had the greatest significance and theoretical direction (40). Once the focused coding of the transcribed

Formatted: Font: 16 pt, Bold

Formatted: Right: 0.25"

interview, field notes, and memos were complete, we performed theoretical coding to conceptualize how the focused codes were related to specific categories (40).

In order to add precision and clarity to our data analysis, theoretical coding was conducted by 1) analyzing and synthesizing relationships between focused codes and 2) building a conceptual model explaining the processes AYACF consider ~~associated~~ with risk perception analysis and decision-making related to dual-method contraceptive use among sexually active AYACF using prescribed contraceptives (40, 48). The development of the conceptual model began during the theoretical coding phase of the first interview and occurred throughout the theoretical coding phase of the remaining interviews. By the thirteenth interview, major themes began to emerge, and were solidified by the twentieth interview. By the twenty-fifth interview data saturation had been achieved as no new themes had emerged and sufficient data was present to support the existing themes within the conceptual model.

Formatted: Font: 16 pt, Bold

~~4.2.6.~~ Trustworthiness and Rigor

To ensure credibility, triangulation was used to compare study findings to the literature throughout the data analysis process. Over the course of the study, data, procedures, and tools were constantly compared against the literature to assess the need for alterations. During the data collection and analysis process, emerging themes were compared to other study findings to assess the need to develop new interview questions to be added to the interview guide. To ensure confirmability, reflexive journaling was conducted after each interview to capture thoughts and perceptions and identify any potential biases.~~-~~ Since the individual conducting the interviews (LH) once identified as an AYACF, reflexive journaling was essential in identifying and

Formatted: Right: 0.25"

processing any potential biases that existed based on prior experiences. To ensure dependability, an audit trail ~~was kept~~ detailing the developmental and implementation stages of this study was kept to verify the accuracy and reliability of the data collected.

Ethical Considerations

~~Permission~~ Approval to conduct this study was obtained from the Duke Institutional Review Board (Pro00111825). While participant risk in this study was minimal, measures were taken to ensure the physical, psychological and emotional well-being of participants were addressed. The sensitive nature of the topic had the potential to reveal incidents of sexual abuse and violence. Thus, measures were in place to report to legal authorities and provide mental health resources had a participant shared an experience of sexual abuse or violence.

Further, to minimize the potential for emotional distress during interviews, participants were encouraged to only share information for which they were comfortable disclosing. If questions or responses lead to emotional distress or discomfort, each participant was provided with an opportunity to pause, reschedule, or stop the interview. To build trust and ensure all participants felt respected in an interview setting, the interviewer (LH) attended and participated in a qualitative research course specific to interviewing participants belonging to structurally oppressed racial and ethnic groups.

The threat to confidentiality was reduced by using a pseudonym or participant code number in place of the participants' names. No actual names of participants were used in the study write-up. During data collection, paper-based sources of data and audio recordings were kept in a secured filing cabinet in a locked office. De-identified electronic data, including transcribed interviews, demographic information and field notes were password-protected and

Formatted: Font: 16 pt, Bold

Formatted: Indent: First line: 0"

Formatted: Font: (Default) +Headings CS (Times New Roman)

Formatted: Right: 0.25"

stored in an encrypted database on a secure Duke University server. Access to both paper-based and electronic databases were limited to the LH (interviewer and coder) and MR (coder).

Formatted: Font: 18 pt, Bold

~~4.3.~~ Results

As ~~demonstrated~~ described in **Table 2**, of the ~~a~~ ~~total of~~ twenty-five AYACF ~~were~~ recruited for this study, ~~;~~ the mean age of participants was 20.7 years and represented diverse racial (African American, 44~~56~~%; White, 4~~0~~%; Asian, 4~~2~~%; ~~Unknown~~Other, 16~~2~~%) and ethnic identities (Hispanic, 48%; Non-Hispanic, 48%; Unknown, 2%). The participants were mainly college-educated (80%) without children (88%) and currently in a monogamous relationship with one male partner (52%). Additionally, ~~the majority of~~most participants had previously been diagnosed with an STI (52%) and were currently using Depo Provera (40%) as their prescribed contraceptive method. Interviews ranged from 18 to 55 minutes, with an average of 38 minutes per interview.

Table 2: Participant Demographics

Age	#	%
18 years old	2	8%
19 years old	3	12%
20 years old	11	44%
21 years old	2	8%
22 years old	2	8%
23 years old	1	4%
24 years old	4	16%

Race/Ethnicity	#	%
White	1	4%
Black	11	44%
Hispanic	8	32%

Formatted: Right: 0.25"

Asian	1	4%
Other	4	16%
**Education**	**#**	**%**
Current College Student	19	76%
Graduated College	1	4%
Non-College	4	16%
High School	1	4%
**Previous STI Diagnosis**	**#**	**%**
Yes	13	52%
No	12	48%
**Birth Control Method**	**#**	**%**
Shot	10	40%
Implant	5	20%
Pill	8	32%
Ring	1	4%
Patch	1	4%
**Relationship Status**	**#**	**%**
Single and having casual sex with one male partner	8	32%
Single and having casual sex with more than one male partner	4	16%
In monogamous relationship with one male partner	13	52%
In open relationship with more than one male partner	0	0%

The data analysis revealed that t~~T~~he process of STI risk perception analysis and dual-method contraceptive decision-making consists of the following overarching themes ~~categories~~ 1) Fear of pregnancy greater than fear of STIs, 2) Making assumptions and partner deferral, and 3) STI Diagnosis: A new reality (Fig ~~ure~~ 1~~. **Dual Method Contraception Decision-Making Factors**~~). For AYACF, SDOH and personal background perpetuate the belief that pregnancy is a more significant concern than STIs. As AYACF navigate sexual relationships, they make assumptions about a sexual partner's STI status based on specific partner characteristics and relationship dynamics. Together, relationship dynamics and partner characteristics influence the

Formatted: Font: Not Bold

Formatted: Right: 0.25"

decision to defer to the sexual partner for dual-method contraceptive decision-making. Additionally, a general lack of concern for STIs contributed to the decision to forgo condom use and rely on STI testing after engaging in sexual intercourse without a condom. As a result of deferring to the sexual partner and substituting condom use for STI testing, an STI diagnosis occurred, causing a shift in STI risk perception, which at times resulted in a behavior change.

~~4.3.1.~~ Fear of Pregnancy > Fear of STIs

Formatted: Font: 16 pt, Bold

For many participants, the fear of an unwanted pregnancy was far greater than their fear of acquiring an STI. Although all participants were prescribed contraceptive users, many reported feeling *scared* and *anxious* about getting pregnant. For example, a twenty-one-year-old Asian college student compared her fear of pregnancy to a feeling of *impending doom*. While the fear of pregnancy was palpable among participants, their fear of STIs was minimal, playing a significant role in their STI risk perception. A twenty-year-old Hispanic college student shared her perspective on pregnancy and STIs:

> *Well, I think pregnancy is definitely a lifetime, like a for-life commitment. And that's definitely way scarier, even though STIs are scary for sure. I don't know. I just feel like when you think of pregnancy, it's like really scary. Like, really scary, I mean, especially for women. I've always just been scared of my life stopping there.*

Having an overwhelming fear of unwanted pregnancy contributed to dual-method contraceptive decision-making as several participants stopped using condoms once initiating a prescribed contraceptive. For example, a twenty-year-old Hispanic college student with a previous STI diagnosis described her experience of using condoms before initiating prescribed contraceptive use:

Formatted: Right: 0.25"

So I have used a condom since, like, I started the first time [being sexually active]. *And I've been using a condom since then until I went on birth control for the whole time, and then I stopped using a condom.*

Further, participants who engaged in dual-method contraceptive use reported using condoms for additional pregnancy protection as opposed to STI and HIV protection. A twenty-four-year-old Hispanic college student explained why she used condoms in addition to a prescribed contraceptive.

I think it was mostly, like, my thinking of condoms was, like, me just not getting pregnant. It was, like, another barrier for birth control, basically.

~~4.3.2.~~ Social Determinants of Health

Formatted: Font: 16 pt, Bold

Recent changes to Texas abortion laws exacerbated participants' fear of unwanted pregnancy. As a result of newly implemented state laws banning abortion, participants considered additional ways to prevent pregnancy and detect pregnancy at the earliest stage possible. In the quote below, a nineteen-year-old African American college student shared her experience:

I feel like this makes me more conscious of knowing, like, I need to know right now if I think I'm pregnant because we should not be wasting time.

Further, participants contemplated how they could access or self-induce an abortion in the instance that they became pregnant. For example, a nineteen-year-old college student who identified as Hispanic, African American, and White shared how she would approach an unplanned pregnancy.

Formatted: Right: 0.25"

> *I'm pretty confident that I would catch it* [pregnancy] *in time, and I will just get mailed in some abortion pills and just take that and naturally miscarry.*

A twenty-one-year-old Asian college student shared a similar perspective on what she would do if she were to experience an unplanned pregnancy.

> *I can't afford an abortion. Do I have to buy a bunch of vitamin C? To have like one of those, like self-abortions or whatever I hear people talking about like a hanger.*

School-based sex education also played a significant role in how participants perceived pregnancy and STIs. For example, a twenty-one-year-old Asian college student shared her sex education experience:

> *They just instill a lot of fear of not getting pregnant at an early age and not getting STDs. And it's, it's effective because I'm deadly scared of getting pregnant at my age.*

The majority of participants received a sexual health education in school that primarily focused on abstinence. A nineteen-year-old African American college student with a prior STI diagnosis said:

> *I'm not gonna lie, like when I was younger and like Sex-Ed and stuff, they didn't really like teach it. They taught me more like abstain. Because they're like the best way to prevent everything, just abstain.*

~~4.3.4.~~ Personal Background

Formatted: Font: 16 pt, Bold

Breaking the cycle of becoming a mother at a young age was especially important to participants whose mothers had experienced an unplanned pregnancy in their adolescence or early twenties. The hardship experienced by their mothers navigating young motherhood further

Formatted: Right: 0.25"

exacerbated participants’ fear of having an unplanned pregnancy. A twenty-year-old Hispanic college student shared how her mother’s experience influenced her decision to be on a prescribed contraceptive.

My mom's experiences have also had a big impact on how breaking the cycle is actually important and how it's up to you whether you want to be better for yourself and for your family.

Similarly, a twenty-year-old Hispanic college student shared how her mother’s openness about her own life experiences shaped her contraceptive decision-making.

…my mom is very open to her past life or her childhood, and she talks about how those experiences have like made her feel, and it just kind of like impacts me because…I don't want to like I guess disappoint her in the way where it's like I'm gonna do the same or like all her sacrifices are for nothing.

Relationships between participants and their mothers played a major role in how participants perceived STI risk. For example, participants who talked to their mothers about sex-related topics such as pregnancy and STI prevention methods were less likely to practice dual-method contraceptive use and more likely to have had a previous STI diagnosis compared to participants who have openly discussed sex-related topics with their mother. Below, a nineteen-year-old college student with a prior STI diagnosis who identified as African-American, Hispanic, and White shared her experience of attempting to discuss sex and pregnancy prevention with her mother.

And my mom she just kind of was always kind of like, was kind of shushing me. She was like, oh no, like don't have sex like, I don't want to, I don't want to know about that, you

Formatted: Right: 0.25"

have to be a virgin. Like she, she wanted, I think she would prefer if I was a virgin until I'm like 20, 23 or something.

~~4.3.5.~~ Making Assumptions: Participant Characteristics, Relationship Dynamic, and Defer to Partner

Formatted: Font: 16 pt, Bold

Instead of using condoms or inquiring about condom use, participants made assumptions about their partner's STI status based on specific partner characteristics. For example, age played a significant role in STI risk perception analysis and dual-method contraceptive decision-making as participants with older sexual partners were more likely to assume that their partner did not have an STI, resulting in less dual-method contraceptive use. Older partners were *mature* and *responsible*. However, they rarely used condoms or had conversations about condoms or STIs before engaging in sex with the participant. In the quote below, a twenty-two-year-old Hispanic college student recalled a conversation she had with a friend regarding her older sexual partner.

I had mentioned to her [friend] *that I was seeing this older guy. He was 21 at the time. I was like, oh, I really liked him, like, we already have sex, but he tends to pull out. I was like, do you think that's okay?*

Additionally, participants with older partners were more likely to defer to their partners for condom use decision-making. Participants reported that their older partners had more sexual experience, contributing to an uneven power dynamic in the relationship. Below, a twenty-two-year-old Hispanic college student described how she feared being perceived negatively by her older partner if she inquired about condom use.

He was a little bit older than me, and I guess I was shy just because I was like, oh, he's more experienced. Let me not ask these questions: what if he looks down on me?

Formatted: Right: 0.25"

Trustworthiness was another partner characteristic that participants factored into their STI risk perception analysis and dual-method contraceptive decision-making. For example, a twenty-year-old Hispanic college student explained how trust plays a vital role in her dual-method contraceptive use decision-making.

> *I personally don't really use condoms because the people who are my partners are usually people that I trust.*

Meanwhile, several participants attributed a prior STI diagnosis to a partner that they perceived as trustworthy. A nineteen-year-old college student who identified as African American, Hispanic, and White provided insight into a prior STI diagnosis:

> *The first time I ever had condomless sex, I trusted the person because they said they get tested regularly, and that was the first and last time I contracted an STI, which was chlamydia.*

Participants also made assumptions about their partner's STI status based on their partner's sexual history as participants were less likely to use condoms with partners whom they believed to have had a low number of sexual partners. In the quote below, a twenty-year-old Hispanic college student assumed her partner's STI status based on his sexual history:

> *Because I feel like he hasn't been with many. At the top of my head probably like two to three before me, so it wasn't like, oh my god he's been with more than ten. I should probably have him get checked out before or anything like that.*

~~4.3.6.~~ Relationship Dynamic

Formatted: Font: 16 pt, Bold

Formatted: Right: 0.25"

Monogamy, actual or perceived, was a major factor AYACF considered ~~associated~~ with dual-method contraceptive decision-making as participants reported less condom use while being in a perceived monogamous relationship compared to those who were having casual sex. Participants made assumptions about their partner's STI status based on their partners being perceived as *faithful* or *loyal.* Below, a twenty-year-old African American college student reflected on her thought process in a previous relationship.

> *I'm not gonna wear no condom with this person because he's probably never gonna cheat on me or leave me, you know?*

Participants reported not wearing condoms while in non-monogamous sexual relationships or when having sex with a new partner, but not while in a monogamous relationship. For example, a twenty-year-old African American participant with a high school education said, *I just didn't use them* [condoms] *as much because I was in a relationship.*

In some instances, perceived monogamy played a role in participants' decision to defer to their partner for condom use decision-making. For example, an eighteen-year-old Hispanic participant with a high school education reported:

> *He started saying that we were already together, so what was the reason for us to, you know, use condoms if we were just with each other? But I mean, he did cheat on me, so, because obviously I got chlamydia.*

Additionally, condom use was low in relationships where participants reportedly knew their partner for a period of time prior to engaging in sexual intercourse with them. In the quote below, a twenty-four-year-old African American college student provided insight into how familiarity played a role in her dual-method contraceptive decision-making.

Formatted: Right: 0.25"

> *Well, even before then, the person that gave me the last one* [STI], *we used to date back in middle school. So, I felt comfortable with him, you know, it wasn't like some dude off the street.*

~~4.3.7.~~ STI Testing as Reassurance

Formatted: Font: 16 pt, Bold

Frequent STI testing was common among participants who did not practice consistent condom use. Participants often assumed that STI testing (without condom use) was enough to protect them from STIs, Participants reported that STI testing provided them with *reassurance* after engaging in sexual intercourse without a condom. A twenty-four-year-old, Hispanic college graduate discussed her experience:

> *I would just go ahead and book an appointment literally for that week and tell the doctor what was going on, just because I'm very paranoid to some extent, like because I knew I was, like, having casual sex [without a condom].*

Additionally, some participants decided to completely stop wearing condoms because they were getting tested frequently for STIs. In the quote below, a twenty-three-year-old, Hispanic high school graduate with a prior STI diagnosis explained further:

> *Yeah, I'm way more comfortable not using condoms anymore. Yeah, but again, I'm emphasizing like I'm emphasizing getting a screen or getting tested with my partner or making sure that they've been tested recently and then if I do just have sex with them, I make sure I'm like, okay in three months, I'm gonna get tested and I'll see*

Formatted: Right: 0.25"

Participants who continued to have sex with partners who had previously given them an STI preferred STI testing to using condoms. A twenty-four-year-old African American college student shared her experience:

> *Even when, like, when I found out my spouse was cheating, every time we had sex, I set a doctor's appointment, I don't care if it was every week, every two weeks, every three weeks, every month, I always set a doctor's appointment afterwards.*

~~4.3.8.~~ STI Diagnosis: A New Reality

Formatted: Font: 16 pt, Bold

For many participants, an STI diagnosis created a shift in STI risk perception analysis and dual-method contraceptive decision-making. As participants grappled with their STI diagnosis, they began to consider implementing behavioral changes to reduce their likelihood of acquiring another STI in the future. Below, a twenty-three-year-old Hispanic participant with a high school education explained:

> *And that kind of put me in the spot where it's kinda like, oh, I should start watching, you know, watching out who I mess with and who I, I should use more protection.*

Similarly, a twenty-year-old Hispanic, college student shared:

> *So, I was like, in my mind, like, biological, thinking like I can't get pregnant on my period so I didn't really put too much effort into like using a condom, but after chlamydia, I'm like use a condom every time like it's not negotiable.*

Following an STI diagnosis, some participants reported behavior change as they stopped deferring to their partners for condom use decision-making and started demanding condom use

Formatted: Indent: First line: 0.5"

Formatted: Right: 0.25"

from their sexual partners. A twenty-two-year-old Hispanic college student shared her experience:

> *I kind of did put my foot down; I was like, look, it's either we're gonna wear condoms or we're just gonna leave it out here, I was like, I enjoy being with you, obviously, but if you are not gonna follow through with this, then there's no point of us being like that.*

As participants began to take control of their health, they reported experiencing a newfound sense of confidence. For example, a twenty-year-old African American high school graduate said, *When you put your foot down, it feels empowering*. Similarly, a twenty-two-year-old Hispanic college student shared:

> *But you demanding a condom, it's like you know what you want in your life, you take care of yourself, you're aware of what can happen and consequences when having sex.*

Formatted: Indent: First line: 0.5"

~~4.4.~~ Discussion

Formatted: Font: 18 pt, Bold

This grounded theory study provides insight into the complex decision-making processes considered ~~associated~~ with STI risk perception analysis and dual-method contraceptive use among ~~a racially and ethnically diverse group of~~ AYACF in Texas, a population who has experienced an increase in STI rates over the last five years (1)~~F~~. ~~This study~~ We found that AYACF are making assumptions about the STI status of their sexual partners and deferring to them for condom use decision-making. Rather than inquire about their partner's STI status or ask them to wear a condom, participants conducted their own risk perception analysis based on partner characteristics and relationship dynamics.

Formatted: Right: 0.25"

Specifically, perceived trustworthiness and monogamy were the most cited reasons for not practicing dual-method contraceptive use and deferring to partners for condom use decision-making. Further, among participants with a prior STI diagnosis, perceiving their partner as trustworthy and monogamous were the most common reasons for not using a condom during the sexual encounter that led to their STI diagnosis. Our findings coincide with previous studies that found that young adult women who reported high levels of trust and perceived monogamy with a sexual partner were less likely to use a condom during sexual intercourse than those with low levels of trust and perceived monogamy (49, 50).

A ~~A~~ possible explanation for the relationship between perceived trustworthiness and monogamy and partner deferral is that young adult women are more likely to be attached to societal romantic love ideals such as love, faithfulness, and lifelong commitment in their relationship than their male partners, leading them to misjudge trust (51). ~~Additionally,~~ Additionally, partner deferral is more likely to occur in relationships with an uneven power dynamic than in relationships where decision-making and power are equally distributed (52). Such findings were reflected in our study as participants who reported having older and more experienced sexual partners were more likely to defer to them for condom use decision-making.

Patriarchal and societal and cultural norms and expectations is a prevalent and complex issue in the US, and can have devastating emotional, physical, and psychological consequences on girls and young women (53, 54). Future research should focus on developing interventions that help AYACF view males partners from a realistic, as opposed to idealistic, perspective, and increase self-esteem. Concurrently, health care providers can educate young women on the likelihood and reality of acquiring an STI or HIV in relationships where partners are perceived as trustworthy or monogamous.

Formatted: Right: 0.25"

An important finding in this study was the role mothers played in STI risk perception and dual-method contraceptive decision-making. Participants ~~More participants~~ with mothers who avoided sexual health discussions were more likely to have been diagnosed with an STI than participants whose mothers engaged in conversations about sexual health-related topics such as STIs and pregnancy prevention. Further, ~~more~~ participants whose mothers personally experienced or had a close family member experience unintended pregnancy as an adolescent or young adult were more likely to have received information regarding ~~had discussed~~ STI and pregnancy prevention from ~~with~~ their mothors ~~daughters~~ compared to participants with mothers who ~~who~~ had not experienced an unintended pregnancy at a young age.

Mother-daughter communication has been found to reduce ~~reduces~~ STI and unintended pregnancy among adolescents under 18 years of age (55-57). Our findings demonstrate that mother-daughter communication in adolescence may also reduce STI and unintended pregnancy risk in young adulthood. Considering the powerful role mothers played in STI and pregnancy prevention, future research and practice should focus on how to promote and facilitate sexual health communication between mothers and daughters. Additionally, pediatric or gynecological health care appointments can be an opportunity for health care providers to facilitate conversations about contraeption or STIs between females under the age of 18 and their mothers or maternal guardians who accompany them. ~~.~~

Another finding from this study is that participants used STI testing as reassurance after engaging in sexual intercourse without a condom. Conversely, a previous study found that young adult women were using STI testing as a form of reassurance before engaging in sexual intercourse without a condom (10). One possible explanation why this study's~~for~~ participants using STI testing as reassurance is that they ~~participants~~ were recruited from clinics with free

Formatted: Right: 0.25"

sexual health services (including STI testing), eliminating any potential financial barriers to STI testing.

Another possible explanation for participants using STI testing for reassurance after engaging in sexual intercourse without a condom is that participants perceive STIs as temporary illnesses that are treatable or curable, as opposed to chronic. Further, frequent STI testing might be used as a substitue for engaging in condom negotiation with partners prior to sex, a task that could be viewed as uncomfortable or confrontational. While research has demonstrated that increased access to free or affordable health care results in disease reduction (58-60) , there is limited understanding of how having access to free health care influences sexual health decision-making in the United States, a country where the majority of the population do not have access to government-provided health care(61). Future research should focus on the role access to free STI testing plays in dual-method contraceptive decision-making and STI risk perception, and how patients are receiving STI education at clinics that offer free sexual health services.

Additionally, societal factors such as state abortion policy and school-based sex education may have played a significant role in perpetuating fear of unplanned pregnancy among participants. Our study found that recent changes to abortion laws likely exacerbated participants' pre-existing fear of pregnancy as participants reported buying pregnancy tests and Plan B in bulk, as well as considering potentially life-threatening measures to self-induce an abortion after Roe V. Wade was overturned. Meanwhile, almost all participants reported having a sex education that focused on abstinence and avoiding pregnancy but did not provide information about STIs or STI prevention. Thus, their reduced fear of STIs in comparison to pregnancy might be attributed to a lack of knowledge about STIs and their health-related consequences. While it is is known that states with conservative sex education policies have some of the highest STI and

Formatted: Right: 0.25"

abortion rates (prior to June 2022) (62) (63), the relationship between the specific type of sex education curricula provided in public schools (i.e., abstinence-only, abstinence-plus, comprehensive…) and abortion needs to be explored further.

~~4.4.1.~~ Limitations

Formatted: Font: 16 pt, Bold

This study has limitations. First, ~~the majority of~~most participants were in college and identified as Black/African American and/or Hispanic, and therefore the findings may not be applicable to AYACF who do not attend college and/or belong to other racial or ethnic groups. Second, ~~the majority of~~all participants had access to free STI testing and treatment. Thus, findings might not be transferable to populations who do not have free access to contraception nor sexual health care and services. Further, access to free sexual health care might have influenced participants' decision to engage in dual-method contraceptive use. ~~and participants might not have felt as much of a need to protect themselves from STIs as AYACF without access to free STI testing and treatment.~~ Third, this study was cross-sectional, limiting the understanding of the participants' behavior over a period of time. Fourth, all interviews were conducted virtually via Zoom, which could have limited the researcher's ability to interpret participants' overall body language since participants were only viewed from above the waist. Fifth, self-reporting bias could have occurred, as participants potentially felt a need to provide socially "correct" answers pertaining to their sexual health practices (i.e. consistent condom use and conversations with partners about STIs and condom use). Additionally, participants might have felt embarrassed to truthfully share their sexual health practices due to the nature of the subject. Recall bias was, also, a risk as some participants reported on sexual counters that

Formatted: Right: 0.25"

occurred more than three years prior to the interview. Last, the interview guide developed and implemented by the research team was not tested prior to being used in this study. Testing the interview guide prior to study implementation could have validated questions in the interview guide or lead to the development of new questions that more accurately capture factors associated with dual-method contraceptive decision-making or STI risk perception.

~~4.4.2.~~ Conclusion

Formatted: Font: 16 pt, Bold

Our results indicate that dual-method contraceptive decision-making and STI risk perception analysis is a complex process involving personal, relationship, and societal factors. AYACF likely have low STI risk perception, are not practicing dual-method contraceptive use, and are deferring condom use decision-making to partners they perceive as trustworthy or monogamous. Once diagnosed with an STI, AYACF likely stop relying on perceptions and assumptions to determine the need for dual-method contraceptive use and begin to engage in new thinking and behaviors. The shift in perspective and behavior caused by the STI diagnosis highlights the need to empower young women to put their own sexual health needs before their partners.

Additionally, STI education is important in reducing STI risk. Mother-daughter communication may increase dual-method contraceptive use, reducing STI risk. Future research should focus on ways to promote mother-daughter communication about STI prevention in the AYACF population.

Formatted: Right: 0.25"

Formatted: Indent: First line: 0"

References

1. DSHS TDoSHS. Texas STD Surveillance Report: 2022 Annual Report. Texas Department of State Health Services; 2024.
2. CDC CfDCaP. Sexually Transmitted Disease Surveillance 2019. Atlanta Centers for Disease Control and Prevention; 2021.
3. CDC CfDCaP. Teen Birth Rate by State 2022 [Available from: https://www.cdc.gov/nchs/pressroom/sosmap/teen-births/teenbirths.htm.

Formatted: Font: (Default) +Headings CS (Times New Roman), 12 pt

Formatted: Indent: Hanging: 0.5", Line spacing: Double

Formatted: Font: (Default) +Headings CS (Times New Roman), 12 pt

Formatted: Font: (Default) +Headings CS (Times New Roman), 12 pt

Formatted: Right: 0.25"

4. Fuentes L, Ingerick M, Jones R, Lindberg L. Adolescents' and young adults' reports of barriers to confidential health care and receipt of contraceptive services. Journal of Adolescent Health. 2018;62(1):36-43.
5. CDC CfDCaP. Contraception 2020 [Available from: https://www.cdc.gov/reproductivehealth/contraception/index.htm.
6. CDC CfDCaP. Diagnoses of HIV Infection in the United States and Dependent Areas, 2021: Table 7B. Diagnoses of HIV infection attributed to heterosexual contact by year of diagnosis, assigned sex at birth, race/ethnicity and selected characteristics, 2017-2021-United STates and 6 dependent areas: Centers for Disease Control and Prevention; 2023 [Available from: https://www.cdc.gov/hiv/library/reports/hiv-surveillance/vol-34/content/tables.html.
7. CDC CfDCaP. STDs and HIV – CDC Fact Sheet 2021 [Available from: https://www.cdc.gov/std/hiv/stdfact-std-hiv-detailed.htm.
8. Copen CE. Condom Use During Sexual Intercourse Among Women and Men Aged 15-44 in the United States: 2011-2015 National Survey of Family Growth. Natl Health Stat Report. 2017(105):1-18.
9. Martinez GM AJ. Sexual activity and contraceptive use among teenagers aged 15–19 in the United States, 2015–2017. National Center for Health Statistics; 2020.
10. Lemoine J, Teal SB, Peters M, Guiahi M. Motivating factors for dual-method contraceptive use among adolescents and young women: a qualitative investigation. Contraception. 2017;96(5):352-6.
11. Cates WJ, Steiner MJ. Dual Protection Against Unintended Pregnancy and Sexually Transmitted Infections: What Is the Best Contraceptive Approach? Sexually Transmitted Diseases. 2002;29(3):168-74.

Formatted: Font: (Default) +Headings CS (Times New Roman), 12 pt

Formatted: Font: (Default) +Headings CS (Times New Roman), 12 pt

Formatted: Font: (Default) +Headings CS (Times New Roman), 12 pt

Formatted: Font: (Default) +Headings CS (Times New Roman), 12 pt

Formatted: Font: (Default) +Headings CS (Times New Roman), 12 pt

Formatted: Font: (Default) +Headings CS (Times New Roman), 12 pt

Formatted: Right: 0.25"

12. Walsh-Buhi ER, Helmy HL. Trends in long-acting reversible contraceptive (LARC) use, LARC use predictors, and dual-method use among a national sample of college women. Journal of American College Health. 2018;66(4):225-36.
13. Higgins JA, Cooper AD. Dual use of condoms and contraceptives in the USA. Sexual Health. 2012;9(1):73-80.
14. Pazol K, Daniels K, Romero L, Warner L, Barfield W. Trends in Long-Acting Reversible Contraception Use in Adolescents and Young Adults: New Estimates Accounting for Sexual Experience. J Adolesc Health. 2016;59(4):438-42.
15. Garnett C, Pollack L, Rodriguez F, Renteria R, Puffer M, Tebb KP. The Association Between Nonbarrier Contraceptive Use and Condom Use Among Sexually Active Latina Adolescents. Journal of Adolescent Health. 2021;68(5):985-90.
16. Pazol K, Kramer MR, Hogue CJ. Condoms for Dual Protection: Patterns of Use with Highly Effective Contraceptive Methods. Public Health Reports. 2010;125(2):208-17.
17. Sieving RE, Bearinger LH, Resnick MD, Pettingell S, Skay C. Adolescent Dual Method Use: Relevant Attitudes, Normative Beliefs and Self-Efficacy. Journal of Adolescent Health. 2007;40(3):275.e15-.e22.
18. Steiner RJ, Liddon N, Swartzendruber AL, Rasberry CN, Sales JM. Long-Acting Reversible Contraception and Condom Use Among Female US High School Students: Implications for Sexually Transmitted Infection Prevention. JAMA Pediatrics. 2016;170(5):428-34.
19. McNicholas CP, Klugman JB, Zhao Q, Peipert JF. Condom use and incident sexually transmitted infection after initiation of long-acting reversible contraception. American Journal of Obstetrics and Gynecology. 2017;217(6):672.e1-.e6.

Formatted: Right: 0.25"

20. Williams DJ, Noyes JM. How does our perception of risk influence decision-making? Implications for the design of risk information. Theoretical Issues in Ergonomics Science. 2007;8(1):1-35.

21. WHO WHO. Social Determinants of Health: World Health Organization; 2024 [Available from: https://www.who.int/health-topics/social-determinants-of-health#tab=tab_1.

22. WHO WHO. A Conceptual Framework for Action on the Social Determinants of Health 2010 [Available from: https://www.who.int/sdhconference/resources/ConceptualframeworkforactiononSDH_eng.pdf.

23. Lindberg LD, Kantor LM. Adolescents' Receipt of Sex Education in a Nationally Representative Sample, 2011–2019. Journal of Adolescent Health. 2021.

24. Crooks N, Wise A, Frazier T. Addressing sexually transmitted infections in the sociocultural context of black heterosexual relationships in the United States. Social Science & Medicine. 2020;263:113303.

25. Pequegnat W, Stover E. Considering women's contextual and cultural issues in HIV/STD prevention research. Cultural Diversity and Ethnic Minority Psychology. 1999;5(3):287-91.

26. Guttmacher. Sex and HIV Education 2021 [Available from: https://www.guttmacher.org/state-policy/explore/sex-and-hiv-education.

27. Ellison JE, Brown-Podgorski BL, Morgan JR. Changes in Permanent Contraception Procedures Among Young Adults Following the Dobbs Decision. JAMA Health Forum. 2024;5(4):e240424-e.

28. Mitchell JA, Yao M, Maeda R, Lappen JR, Brant AR. Permanent and long-acting reversible contraception volumes at a multihospital system in Ohio before and after Dobbs. Contraception. 2024:110471.

Formatted: Font: (Default) +Headings CS (Times New Roman), 12 pt

Formatted: Font: (Default) +Headings CS (Times New Roman), 12 pt

Formatted: Font: (Default) +Headings CS (Times New Roman), 12 pt

Formatted: Font: (Default) +Headings CS (Times New Roman), 12 pt

Formatted: Font: (Default) +Headings CS (Times New Roman), 12 pt

Formatted: Font: (Default) +Headings CS (Times New Roman), 12 pt

Formatted: Right: 0.25"

29. Qato DM, Myerson R, Shooshtari A, Guadamuz JS, Alexander GC. Use of Oral and Emergency Contraceptives After the US Supreme Court's Dobbs Decision. JAMA Network Open. 2024;7(6):e2418620-e.
30. Gavey N, McPhillips K, Doherty M. "If it's not on, it's not on"—or is it? Discursive constraints on women's condom use. Gender & Society. 2001;15(6):917-34.
31. Jarama SL, Belgrave FZ, Bradford JB, Young M, Honnold JA. Family, cultural and gender role aspects in the context of HIV risk among African American women of unidentified HIV status: An exploratory qualitative study. AIDS Care. 2007;19:307 - 17.
32. Area UWotNC. Gender Roles and Norms: What are they and how do they Affect Children? 2025 [Available from: https://unitedwaynca.org/blog/gender-norms/.
33. Fantasia HC. Influences of Social Norms and Context on Sexual Decision Making Among Adolescent Women. Journal of Midwifery & Women's Health. 2011;56(1):48-53.
34. East L, Jackson D, O'Brien L, Peters K. Condom negotiation: experiences of sexually active young women. J Adv Nurs. 2011;67(1):77-85.
35. Tyler CP, Whiteman MK, Kraft JM, Zapata LB, Hillis SD, Curtis KM, et al. Dual Use of Condoms With Other Contraceptive Methods Among Adolescents and Young Women in the United States. Journal of Adolescent Health. 2014;54(2):169-75.
36. Eisenberg DL, Allsworth JE, Zhao Q, Peipert JF. Correlates of Dual-Method Contraceptive Use: An Analysis of the National Survey of Family Growth (2006–2008). Infectious Diseases in Obstetrics and Gynecology. 2012;2012:717163.
37. Wilson EK, Koo HP. Associations Between Low-Income Women's Relationship Characteristics and Their Contraceptive Use. Perspectives on Sexual and Reproductive Health. 2008;40(3):171-9.

Formatted: Font: (Default) +Headings CS (Times New Roman), 12 pt

Formatted: Font: (Default) +Headings CS (Times New Roman), 12 pt

Formatted: Right: 0.25"

38. Manlove J, Welti K, Karpilow Q. Relationship Violence Typologies and Condom Use in Young Adult Dating Relationships. Perspectives on Sexual and Reproductive Health. 2019;51(2):109-17.

39. Kusunoki Y, Barber JS, Gatny HH, Melendez R. Physical Intimate Partner Violence and Contraceptive Behaviors Among Young Women. J Womens Health (Larchmt). 2018;27(8):1016-25.

40. Charmaz K. Constructing grounded theory: sage; 2014.

41. Bureau USC. QuickFacts: Harris County. 2021.

42. Medicine BCo. Teen Health Clinic. 2021.

43. TDHHS TDoHaHS. Sexually Transmitted Disease in Texas. 2020.

44. Cohen, Crabtree. Qualitative Research Guidelines Project 2006 [Available from: http://www.qualres.org/HomeSemi-3629.html.

45. Bolton M, McKay A, Schneider M. Relational influences on condom use discontinuation: A qualitative study of young adult women in dating relationships. The Canadian Journal of Human Sexuality. 2010;19(3):91.

46. Lumivero. NVivo 12. 2017.

47. Hernandez CA. Theoretical coding in grounded theory methodology. Grounded Theory Review. 2009;8(3).

48. Chun Tie Y, Birks M, Francis K. Grounded theory research: A design framework for novice researchers. SAGE Open Med. 2019;7:2050312118822927-.

49. Caldwell K, Mathews A. The Role of Relationship Type, Risk Perception, and Condom Use in Middle Socioeconomic Status Black Women's HIV-prevention Strategies. J Black Sex Relatsh. 2015;2(2):91-120.

Formatted: Font: (Default) +Headings CS (Times New Roman), 12 pt

Formatted: Font: (Default) +Headings CS (Times New Roman), 12 pt

Formatted: Font: (Default) +Headings CS (Times New Roman), 12 pt, Portuguese (Brazil)

Formatted: Right: 0.25"

50. Godinho CA, Pereira CR, Pegado A, Luz R, Alvarez MJ. Condom use across casual and committed relationships: The role of relationship characteristics. PLoS One. 2024;19(7):e0304952.

51. Meier A, Hull KE, Ortyl TA. Young Adult Relationship Values at the Intersection of Gender and Sexuality. Journal of Marriage and Family. 2009;71(3):510-25.

52. Woolf SE, Maisto SA. Gender Differences in Condom Use Behavior? The Role of Power and Partner-Type. Sex Roles. 2008;58:689-701.

53. Chan E, Catabay CJ, Campbell JC, Rudolph AE, Stockman JK, Tsuyuki K. Feminine gender norms and syndemic harmful drinking, sexual violence, and sexually transmitted infections among Black women at risk for HIV. Drug Alcohol Depend. 2021;221:108566.

54. Brady J, Iwamoto DK, Grivel M, Kaya A, Clinton L. A systematic review of the salient role of feminine norms on substance use among women. Addictive behaviors. 2016;62:83-90.

55. Teitelman AM, Calhoun J, Duncan R, Washio Y, McDougal R. Young women's views on testing for sexually transmitted infections and HIV as a risk reduction strategy in mutual and choice-restricted relationships. Appl Nurs Res. 2015;28(3):215-21.

56. Teitelman AM, Ratcliffe SJ, Cederbaum JA. Parent—Adolescent Communication About Sexual Pressure, Maternal Norms About Relationship Power, and STI/HIV Protective Behaviors of Minority Urban Girls. Journal of the American Psychiatric Nurses Association. 2008;14(1):50-60.

57. Hutchinson M. The Influence of Sexual Risk Communication Between Parents and Daughters on Sexual Risk Behaviors*. Family Relations. 2004;51:238-47.

58. Zhang X, Bullard KM, Gregg EW, Beckles GL, Williams DE, Barker LE, et al. Access to Health Care and Control of ABCs of Diabetes. Diabetes Care. 2012;35(7):1566-71.

Formatted: Font: (Default) +Headings CS (Times New Roman), 12 pt

Formatted: Right: 0.25"

59. Christopher AS, McCormick D, Woolhandler S, Himmelstein DU, Bor DH, Wilper AP. Access to Care and Chronic Disease Outcomes Among Medicaid-Insured Persons Versus the Uninsured. Am J Public Health. 2016;106(1):63-9.

60. Alcalá HE, Albert SL, Roby DH, Beckerman J, Champagne P, Brookmeyer R, et al. Access to Care and Cardiovascular Disease Prevention: A Cross-Sectional Study in 2 Latino Communities. Medicine (Baltimore). 2015;94(34):e1441.

61. Katherine Keisler-Starkey LNB, and Rachel A. Lindstrom. U.S. Census Bureau, Current Population Reports Washington, DC: U.S. Government Publishing Office; 2023 [Available from: https://www.census.gov/library/publications/2023/demo/p60-281.html?utm_source=chatgpt.com.

62. Guttmacher. Sex and HIV Education Guttmacher Institute2023 [updated 1 July 2023. Available from: https://www.guttmacher.org/state-policy/explore/sex-and-hiv-education.

63. KFF. Rate of Legal Abortions per 1,000 Women Aged 15-44 Years by State of Occurrence 2021 [Available from: https://www.kff.org/womens-health-policy/state-indicator/abortion-rate/?currentTimeframe=1&sortModel=%7B%22colId%22:%22Abortion%20Rate%22,%22sort%22:%22desc%22%7D.

Formatted: Font: (Default) +Headings CS (Times New Roman), 12 pt

Formatted: Font: (Default) +Headings CS (Times New Roman), 12 pt

Formatted: Font: (Default) +Headings CS (Times New Roman), 12 pt

Formatted: Font: (Default) +Headings CS (Times New Roman), 12 pt

Formatted: Font: (Default) +Headings CS (Times New Roman), 12 pt

Formatted: Font: (Default) +Headings CS (Times New Roman), 12 pt

Formatted: Indent: Hanging: 0.5"

Formatted: Right: 0.25"

Formatted: Right: 0.25"

Response to Reviewers

Dear editors of *PLOS ONE,*

Thank you for the opportunity to revise our manuscript, “The Theoretical Understanding of Risk Perception and Dual-Method Contraceptive Decision-Making among Texas Adolescent and Young Adult Women” (PONE-D-24-49168).

We have provided responses to each of the questions from the two reviewers below.

We would be happy to provide any additional information that may be needed.

Sincerely,

Lauren Holt

Reviewer 1

- The introduction section is quite lengthy and could be condensed to focus more directly on the study objectives and rationale. Consider moving some of the background information on STI rates and contraceptive use to a separate background section.

  Response: Thank you for this feedback. The Introduction has been condensed, and a Background section has been added (Lines 48-180)

- Consider adding a separate "Theoretical Framework" section to explain the grounded theory approach in more detail, as this is central to the study methodology.

  Response: Thank you for this feedback. More detail regarding the theoretical framework has been provided in the Study Design section (Lines 183-249).

- The research questions/aims could be stated more explicitly early in the introduction. Currently they are embedded in the "Study Purpose" subsection.

  Response: Thank you for this suggestion. The Introduction section has been condensed allowing the purpose of the study to be stated sooner. (Lines 40-47)

- Provide more details on the data analysis procedures used, including the specific coding approach and how themes were developed.

  Response: Thank you for this suggestion. More information regarding the data analysis procedures has been provided in (Lines 347-379)

- Include information on how many participants were recruited through each method (clinic vs. referral).

Response: Thank you for making this suggestion. Information has been provided regarding how many participants were recruited through each method (clinic vs. referral) (Lines 303-316)

- Clarify whether data saturation was reached and how this was determined.

  Response: Thank you for this feedback. We have explained how we achieved data saturation in Lines 307-320

- Explain the rationale for conducting interviews virtually rather than in-person and discuss any potential limitations of this approach.

  Response: Thank you for making this suggestion. Further information regarding the decision to interview participants virtually is provided in lines 318-320, and limitations were discussed in lines 769-771.

- Provide more information on how trustworthiness and rigor were ensured in the data collection and analysis process

  Response: Thank you for your feedback. More information has been provided regarding the trustworthiness and rigor conducted throughout the study. (Lines 380-391)

- Consider discussing potential limitations of the single-coder approach to data analysis.

  Response: Thank you for this feedback. A two-coder approach was used for this study. More clarity has been provided on this approach in the Data Analysis section (Lines 335-379).

- Provide more specific recommendations for practice and future research based on the study findings.

  Response: Thank you for this suggestion. More specific recommendations for practice and future research have been provided in the Discussion section (Lines 696-700 , Lines 756-760, Lines 712-774)

- Discuss the transferability of the findings to other contexts or populations.

  Response: Thank you for this feedback. Transferability is discussed further in the Limitations section (Lines 762-768)

Reviewer 2

- The abstract should be able to "standalone". The methods sections is inadequate. How many participants were included? Sampling method and data analysis?

  Response: Thank you for this feedback. The abstract has been modified to include a more detail regarding the methods used to conduct the study.

- The manuscript addresses a highly significant public health issue—STIs, HIV risk, and contraceptive use—which is a pressing concern in Texas and nationally. It provides a valuable and timely exploration of STI and HIV risk, contraceptive use, and disparities among AYACF. However, it would benefit from reducing redundancy, clarifying its focus, integrating sections more cohesively, and providing clear research objectives. Addressing these weaknesses will enhance its impact and readability.

  Response: Thank you for this suggestion. The Introduction section has been condensed and re-formatted to facilitate readability.

- Certain statements, such as the role of risk perception in dual-method contraceptive use, are repeated excessively (e.g., paragraph 4 and paragraph 6).

  Response: Thank you for this feedback. Repetitive statements have been removed from these paragraphs.

- The section on *Roe v. Wade* and the subsequent shift in contraceptive behaviours, while relevant, feels somewhat disconnected from the core discussion of STI/HIV risk and dual-method contraceptive use.

  Response: Thank you for this feedback. The Background section has been re-organized to better incorporate the Roe. V. Wade section. (Lines 123-129)

- There is a mention of "societal and cultural norms and values also play an integral role," it does not sufficiently elaborate on how these norms directly shape decision-making processes for AYACF. Provide specific examples or data illustrating how societal expectations (e.g., stigma around condom use or contraception) influence behaviour.

  Response: Thank you for this feedback. More information has been provided in Lines 130-168

- While statistics are valuable, the manuscript sometimes lacks contextualization. For example, stating that "23% of AYACF use condoms during every sexual encounter" could benefit from a comparison to other demographic groups or historical trends to underscore the significance of this figure. Supplement raw statistics with interpretative commentary to help the reader understand their implications.

  Response: Thank you for this feedback. A comparison has been provided for this statistic. (Lines 20-21)

- The gaps in understanding STI and HIV risk perception and dual-method contraceptive decision-making are highlighted, but there is no clear articulation of the research questions or hypotheses driving this investigation. End the introduction with a clear statement of purpose, objectives, or research questions.

Response: Thank you for this suggestion. The introduction has been condensed, allowing for the Study Purpose section to be brought to the readers' attention at an earlier point in the manuscript.

- Certain sentences are overly complex, making them harder to follow (e.g., "The existing age, gender, racial, and ethnic STI and HIV disparities highlight the need to consider how social determinants influence dual-method contraceptive use among AYACF").

  Response: Thank you for this suggestion. Changes have been made to make sentences more clear and concise.

- The manuscript effectively identifies disparities and contributing factors but lacks a preliminary discussion of potential interventions or strategies to address the issues highlighted.

  Response: Thank you for this feedback. Given the grounded theory approach, the research team did not feel current findings would allow for discussion of meaningful potential interventions or strategies at this phase, but there are active plans to build on this research to allow us the ability to do so in the future.

- There is a mention of "study purpose" in 4.1.2 but then the researchers discuss the objectives of the study instead. There is also "aim 1 and 2", it is not clear why there is more than one aim. Please revise.

  Response: Thank you for this feedback. The Introduction now only includes the study purpose to reduce confusion. (Lines 40-47)

- Include more detail about how reflexivity was actively used to enhance study quality would strengthen the discussion.

  Response: Thank you for this suggestion. More detail regarding reflexivity has been included. (Lines 381-391)

- While theoretical sampling is appropriate, the manuscript does not justify why 25 participants were sufficient to achieve saturation. A brief discussion of how saturation was determined could enhance transparency.

  Response: Thank you for this feedback. More detail regarding data saturation has been provided. (Lines 307-320).

- Recruiting participants in person at a health clinic or through referrals may limit the diversity of perspectives, as it excludes AYACF who do not access clinic services. This could bias findings toward individuals with better access to care.

  Response: Thank you for this feedback. This issue has been addressed in the Limitations section of the Discussion section. (Lines 764-768)

- Although interview questions were developed by a research team specializing in sexual and reproductive health, the manuscript does not specify if pilot testing or validation of the interview guide was conducted before data collection. This omission raises concerns about the reliability of the guide.

  Response: Thank you for this important feedback. A pilot test of the interview guide was not performed, and has been addressed in our Limitations section (Lines 785-789)

- The addition of questions after the 14th interview is described as part of the triangulation process, but it is unclear how this was justified methodologically or whether these changes introduced inconsistencies across interviews.

  Response: Thank you for this feedback. More information regarding the insertion of additional questions has been provided. (Lines 273-289).

- While consent is mentioned, there is no explicit discussion of how ethical approval was obtained, how participant confidentiality was maintained (beyond encrypted recording), or how potentially sensitive topics (e.g., relationship violence) were addressed.

  Response: Thank you for this feedback. Information regarding ethical considerations have been provided in the Methodology section (Lines 392-417).

- Given the emphasis on racial and ethnic diversity, the methods lack detail on how interviews accounted for cultural sensitivity or if interviewers were trained in cultural competence

  Response: Thank you for this feedback. Information regarding cultural competence and sensitivity has been provided in Lines 407-410.

- Although NVivo is mentioned as a tool for analysis, there is no discussion of how codes were generated, refined, or validated by the research team. Including examples of codebooks or inter-rater reliability assessments would bolster methodological rigor.

  Response: Thank you for this feedback. More information regarding the coding process has been provided. (Lines 347-379)

- Include a discussion on how data saturation was determined and whether 25 interviews were sufficient to explore diverse experiences thoroughly.

  Response: Thank you for this feedback. We have explained how we achieved data saturation in lines 307-320

- Acknowledge potential biases in recruitment and discuss their implications for the study findings.

  Response: Thank you for this suggestion. This issue has been addressed in the Limitations section in the Discussion (Lines 762-789)

- Include a description of the coding process, how codes were refined, and how consensus was reached among researchers.

Response: Thank you for this feedback. More information regarding the coding process has been provided. (Lines 347-379)

- Table 2 contains discrepancies between the written description of demographics and the tabulated data. For instance, racial/ethnic breakdowns in Table 2 do not match those described in the text (e.g., White: 40% vs. 4%; African American: 56% vs. 44%).

  Response: Thank you for pointing out this error. Changes have been made to ensure the written description and table are consistent.

- The study focuses on AYACF, but the results do not address potential selection biases or whether findings are generalizable beyond this group.

  Response: Thank you for this feedback. Generalizability has been discussed further in the Limitations section (lines 762-789).

- The text mentions "Figure 1: Dual Method Contraception Decision-Making Factors," but the figure is absent.

  Response: The figure was originally submitted in a separate document per PLOS One requirements. It remains in an additional document labelled "Fig 1".

- Some quotes reiterate similar points (e.g., fear of pregnancy, partner trustworthiness) without adding new insights. Consolidate repetitive examples and focus on unique perspectives to avoid redundancy.

  Response: Thank you for this feedback. Although some quotes intersect, each one is meant to serve as supporting evidence for a specific theme identified in the concept model (Fig 1).

- Emphasize the diversity in AYACF experiences and caution against generalizing findings to all young adults, particularly those from different socioeconomic or cultural backgrounds.

  Response: Thank you for this feedback. Please see lines 636-638.

- Statements like "mother-daughter communication reduces STI and unintended pregnancy" imply causation, which cannot be established from cross-sectional data. Reframe such conclusions to reflect correlations, e.g., "Our findings suggest a potential link between mother-daughter communication and reduced STI and unintended pregnancy risk."

  Response: Thank you for this feedback. This sentence refers to findings from previous studies and has been reframed. (Lines 664-667).

- While some limitations are acknowledged, others are overlooked, such as potential biases in self-reported data and the influence of clinic recruitment settings. Expand the limitations section to address self-reporting bias, recall bias, and the specific impact of recruiting participants from clinics offering free services.

Response: Thank you for this suggestion. The Limitations section has been expanded. (Lines 762-789).

- The discussion mentions societal influences like abortion policies and abstinence-based sex education but does not explore their interplay in detail.

  Response: Thank you for this suggestion. The interplay of abstinence-based sex education and abortion policies have been discussed further in Lines 756-760

- While the study involves racially and ethnically diverse participants, there is little discussion of how intersecting factors (e.g., race, ethnicity, socioeconomic status) shape their experiences.

  Response: Thank you for these important comments. We agree that intersectional analysis of key factors would provide add more dimension to the understanding of the experience however the intersecting factors related to race and ethnicity were not found to be significant enough to address. You comment will be very helpful for the research team as we develop next steps to build on this research.

- Expand on why trust and STI testing as reassurance are prevalent among AYACF. Are these influenced by cultural norms, perceived stigma, or misinformation about STIs?

  Response: Thank you for this suggestion. More information about the relationship between trust and STI testing as reassurance has been provided. (Lines 671-700).

- Recommendations for future research are somewhat broad (e.g., promoting mother-daughter communication).

  Response: Thank you for this suggestion. Please see lines (Lines 694-700; 712-724; 735-739; 756-760).

- While the findings are insightful, practical applications for healthcare providers, educators, or policymakers are not sufficiently detailed. Provide actionable recommendations for interventions, such as educational campaigns, clinic-based counselling, or school programs addressing STI prevention.

  Response: Thank you for this feedback. More detailed research and applications have been further discussed in the Discussion section. (Lines 694-700; 712-724; 735-739; 756-760).
