# Supplementary material for: The theoretical understanding of risk perception and dual-method contraceptive decision-making among Texas adolescent and young adult cisgender females
Source: PLoS One. 2026 Apr 1;21(4):e0343687. doi: 10.1371/journal.pone.0343687 (PMC13042665; doi:10.1371/journal.pone.0343687)
Supplement: Attachment — Submitted filename: Review of submission PONE-D-24-49168.docx [file pone.0343687.s001.docx]

**Structure and Organization**

- The introduction section is quite lengthy and could be condensed to focus more directly on the study objectives and rationale. Consider moving some of the background information on STI rates and contraceptive use to a separate background section.
- The methods section would benefit from clearer subheadings to delineate the different components (e.g., Study Design, Participants, Data Collection, Data Analysis).
- Consider adding a separate "Theoretical Framework" section to explain the grounded theory approach in more detail, as this is central to the study methodology.

**Content and Clarity**

- The research questions/aims could be stated more explicitly early in the introduction. Currently they are embedded in the "Study Purpose" subsection.
- Provide more details on the data analysis procedures used, including the specific coding approach and how themes were developed.
- Include information on how many participants were recruited through each method (clinic vs. referral).
- Clarify whether data saturation was reached and how this was determined.

**Methodological Considerations**

- Explain the rationale for conducting interviews virtually rather than in-person and discuss any potential limitations of this approach.
- Provide more information on how trustworthiness and rigor were ensured in the data collection and analysis process.
- Consider discussing potential limitations of the single-coder approach to data analysis.

**Discussion and Implications**

- Expand the discussion of how the findings relate to existing literature and theory on contraceptive decision-making.
- Provide more specific recommendations for practice and future research based on the study findings.
- Discuss the transferability of the findings to other contexts or populations.
