# Supplementary material for: The theoretical understanding of risk perception and dual-method contraceptive decision-making among Texas adolescent and young adult cisgender females
Source: PLoS One. 2026 Apr 1;21(4):e0343687. doi: 10.1371/journal.pone.0343687 (PMC13042665; doi:10.1371/journal.pone.0343687)
Supplement: Attachment — Submitted filename: Response to Reviewers_PLOSONE_23May2025.docx [file pone.0343687.s005.docx]

Response to Reviewers

Dear editors of *PLOS ONE,*

Thank you for the opportunity to revise our manuscript, “The Theoretical Understanding of Risk Perception and Dual-Method Contraceptive Decision-Making among Texas Adolescent and Young Adult Women” (PONE-D-24-49168).

We have provided responses to each of the questions from the two reviewers below.

We would be happy to provide any additional information that may be needed.

Sincerely,

Lauren Holt

**Reviewer 1**

- The introduction section is quite lengthy and could be condensed to focus more directly on the study objectives and rationale. Consider moving some of the background information on STI rates and contraceptive use to a separate background section.

**Response:** Thank you for this feedback. The Introduction has been condensed, and a Background section has been added (Lines 48-180)

- Consider adding a separate "Theoretical Framework" section to explain the grounded theory approach in more detail, as this is central to the study methodology.

**Response:** Thank you for this feedback. More detail regarding the theoretical framework has been provided in the Study Design section (Lines 183-249).

- The research questions/aims could be stated more explicitly early in the introduction. Currently they are embedded in the "Study Purpose" subsection.

**Response:** Thank you for this suggestion. The Introduction section has been condensed allowing the purpose of the study to be stated sooner. (Lines 40-47)

- Provide more details on the data analysis procedures used, including the specific coding approach and how themes were developed.

**Response:** Thank you for this suggestion. More information regarding the data analysis procedures has been provided in (Lines 347-379)

- Include information on how many participants were recruited through each method (clinic vs. referral).

**Response:** Thank you for making this suggestion. Information has been provided regarding how many participants were recruited through each method (clinic vs. referral) (Lines 303-316)

- Clarify whether data saturation was reached and how this was determined.

**Response:** Thank you for this feedback. We have explained how we achieved data saturation in Lines 307-320

- Explain the rationale for conducting interviews virtually rather than in-person and discuss any potential limitations of this approach.

**Response:** Thank you for making this suggestion. Further information regarding the decision to interview participants virtually is provided in lines 318-320, and limitations were discussed in lines 769-771.

- Provide more information on how trustworthiness and rigor were ensured in the data collection and analysis process

**Response:** Thank you for your feedback. More information has been provided regarding the trustworthiness and rigor conducted throughout the study. (Lines 380-391)

- Consider discussing potential limitations of the single-coder approach to data analysis.

**Response:** Thank you for this feedback. A two-coder approach was used for this study. More clarity has been provided on this approach in the Data Analysis section (Lines 335-379).

- Provide more specific recommendations for practice and future research based on the study findings.

**Response:** Thank you for this suggestion. More specific recommendations for practice and future research have been provided in the Discussion section (Lines 696-700 , Lines 756-760, Lines 712-774)

- Discuss the transferability of the findings to other contexts or populations.

**Response:** Thank you for this feedback. Transferability is discussed further in the Limitations section (Lines 762-768)

**Reviewer 2**

- The abstract should be able to “standalone”. The methods sections is inadequate. How many participants were included? Sampling method and data analysis?

**Response:** Thank you for this feedback. The abstract has been modified to include a more detail regarding the methods used to conduct the study.

- The manuscript addresses a highly significant public health issue—STIs, HIV risk, and contraceptive use—which is a pressing concern in Texas and nationally. It provides a valuable and timely exploration of STI and HIV risk, contraceptive use, and disparities among AYACF. However, it would benefit from reducing redundancy, clarifying its focus, integrating sections more cohesively, and providing clear research objectives. Addressing these weaknesses will enhance its impact and readability.

**Response:** Thank you for this suggestion. The Introduction section has been condensed and re-formatted to facilitate readability.

- Certain statements, such as the role of risk perception in dual-method contraceptive use, are repeated excessively (e.g., paragraph 4 and paragraph 6).

**Response:** Thank you for this feedback. Repetitive statements have been removed from these paragraphs.

- The section on *Roe v. Wade* and the subsequent shift in contraceptive behaviours, while relevant, feels somewhat disconnected from the core discussion of STI/HIV risk and dual-method contraceptive use.

**Response:** Thank you for this feedback. The Background section has been re-organized to better incorporate the Roe. V. Wade section. (Lines 123-129)

- There is a mention of "societal and cultural norms and values also play an integral role," it does not sufficiently elaborate on how these norms directly shape decision-making processes for AYACF. Provide specific examples or data illustrating how societal expectations (e.g., stigma around condom use or contraception) influence behaviour.

**Response:** Thank you for this feedback. More information has been provided in Lines 130-168

- While statistics are valuable, the manuscript sometimes lacks contextualization. For example, stating that "23% of AYACF use condoms during every sexual encounter" could benefit from a comparison to other demographic groups or historical trends to underscore the significance of this figure. Supplement raw statistics with interpretative commentary to help the reader understand their implications.

**Response:** Thank you for this feedback. A comparison has been provided for this statistic. (Lines 20-21)

- The gaps in understanding STI and HIV risk perception and dual-method contraceptive decision-making are highlighted, but there is no clear articulation of the research questions or hypotheses driving this investigation. End the introduction with a clear statement of purpose, objectives, or research questions.

**Response:** Thank you for this suggestion. The introduction has been condensed, allowing for the Study Purpose section to be brought to the readers’ attention at an earlier point in the manuscript.

- Certain sentences are overly complex, making them harder to follow (e.g., "The existing age, gender, racial, and ethnic STI and HIV disparities highlight the need to consider how social determinants influence dual-method contraceptive use among AYACF").

**Response:** Thank you for this suggestion. Changes have been made to make sentences more clear and concise.

- The manuscript effectively identifies disparities and contributing factors but lacks a preliminary discussion of potential interventions or strategies to address the issues highlighted.

**Response:** Thank you for this feedback. Given the grounded theory approach, the research team did not feel current findings would allow for discussion of meaningful potential interventions or strategies at this phase, but there are active plans to build on this research to allow us the ability to do so in the future.

- There is a mention of “study purpose” in 4.1.2 but then the researchers discuss the objectives of the study instead. There is also “aim 1 and 2”, it is not clear why there is more than one aim. Please revise.

**Response:** Thank you for this feedback. The Introduction now only includes the study purpose to reduce confusion. (Lines 40-47)

- Include more detail about how reflexivity was actively used to enhance study quality would strengthen the discussion.

**Response:** Thank you for this suggestion. More detail regarding reflexivity has been included. (Lines 381-391)

- While theoretical sampling is appropriate, the manuscript does not justify why 25 participants were sufficient to achieve saturation. A brief discussion of how saturation was determined could enhance transparency.

**Response:** Thank you for this feedback. More detail regarding data saturation has been provided. (Lines 307-320).

- Recruiting participants in person at a health clinic or through referrals may limit the diversity of perspectives, as it excludes AYACF who do not access clinic services. This could bias findings toward individuals with better access to care.

**Response:** Thank you for this feedback. This issue has been addressed in the Limitations section of the Discussion section. (Lines 764-768)

- Although interview questions were developed by a research team specializing in sexual and reproductive health, the manuscript does not specify if pilot testing or validation of the interview guide was conducted before data collection. This omission raises concerns about the reliability of the guide.

**Response:** Thank you for this important feedback. A pilot test of the interview guide was not performed, and has been addressed in our Limitations section (Lines 785-789)

- The addition of questions after the 14th interview is described as part of the triangulation process, but it is unclear how this was justified methodologically or whether these changes introduced inconsistencies across interviews.

**Response:** Thank you for this feedback. More information regarding the insertion of additional questions has been provided. (Lines 273-289).

- While consent is mentioned, there is no explicit discussion of how ethical approval was obtained, how participant confidentiality was maintained (beyond encrypted recording), or how potentially sensitive topics (e.g., relationship violence) were addressed.

**Response:** Thank you for this feedback. Information regarding ethical considerations have been provided in the Methodology section (Lines 392-417).

- Given the emphasis on racial and ethnic diversity, the methods lack detail on how interviews accounted for cultural sensitivity or if interviewers were trained in cultural competence

**Response:** Thank you for this feedback. Information regarding cultural competence and sensitivity has been provided in Lines 407-410.

- Although NVivo is mentioned as a tool for analysis, there is no discussion of how codes were generated, refined, or validated by the research team. Including examples of codebooks or inter-rater reliability assessments would bolster methodological rigor.

**Response:** Thank you for this feedback. More information regarding the coding process has been provided. (Lines 347-379)

- Include a discussion on how data saturation was determined and whether 25 interviews were sufficient to explore diverse experiences thoroughly.

**Response:** Thank you for this feedback. We have explained how we achieved data saturation in lines 307-320

- Acknowledge potential biases in recruitment and discuss their implications for the study findings.

**Response:** Thank you for this suggestion. This issue has been addressed in the Limitations section in the Discussion (Lines 762-789)

- Include a description of the coding process, how codes were refined, and how consensus was reached among researchers.

**Response:** Thank you for this feedback. More information regarding the coding process has been provided. (Lines 347-379)

- Table 2 contains discrepancies between the written description of demographics and the tabulated data. For instance, racial/ethnic breakdowns in Table 2 do not match those described in the text (e.g., White: 40% vs. 4%; African American: 56% vs. 44%).

**Response:** Thank you for pointing out this error. Changes have been made to ensure the written description and table are consistent.

- The study focuses on AYACF, but the results do not address potential selection biases or whether findings are generalizable beyond this group.

**Response:** Thank you for this feedback. Generalizability has been discussed further in the Limitations section (lines 762-789).

- The text mentions "Figure 1: Dual Method Contraception Decision-Making Factors," but the figure is absent.

**Response:** The figure was originally submitted in a separate document per PLOS One requirements. It remains in an additional document labelled “Fig 1”.

- Some quotes reiterate similar points (e.g., fear of pregnancy, partner trustworthiness) without adding new insights. Consolidate repetitive examples and focus on unique perspectives to avoid redundancy.

**Response:** Thank you for this feedback. Although some quotes intersect, each one is meant to serve as supporting evidence for a specific theme identified in the concept model (Fig 1).

- Emphasize the diversity in AYACF experiences and caution against generalizing findings to all young adults, particularly those from different socioeconomic or cultural backgrounds.

**Response:** Thank you for this feedback. Please see lines 636-638.

- Statements like "mother-daughter communication reduces STI and unintended pregnancy" imply causation, which cannot be established from cross-sectional data. Reframe such conclusions to reflect correlations, e.g., "Our findings suggest a potential link between mother-daughter communication and reduced STI and unintended pregnancy risk."

**Response:** Thank you for this feedback. This sentence refers to findings from previous studies and has been reframed. (Lines 664-667).

- While some limitations are acknowledged, others are overlooked, such as potential biases in self-reported data and the influence of clinic recruitment settings. Expand the limitations section to address self-reporting bias, recall bias, and the specific impact of recruiting participants from clinics offering free services.

**Response:** Thank you for this suggestion. The Limitations section has been expanded. (Lines 762-789).

- The discussion mentions societal influences like abortion policies and abstinence-based sex education but does not explore their interplay in detail.

**Response:** Thank you for this suggestion. The interplay of abstinence-based sex education and abortion policies have been discussed further in Lines 756-760

- While the study involves racially and ethnically diverse participants, there is little discussion of how intersecting factors (e.g., race, ethnicity, socioeconomic status) shape their experiences.

**Response:** Thank you for these important comments. We agree that intersectional analysis of key factors would provide add more dimension to the understanding of the experience however the intersecting factors related to race and ethnicity were not found to be significant enough to address. You comment will be very helpful for the research team as we develop next steps to build on this research.

- Expand on why trust and STI testing as reassurance are prevalent among AYACF. Are these influenced by cultural norms, perceived stigma, or misinformation about STIs?

**Response:** Thank you for this suggestion. More information about the relationship between trust and STI testing as reassurance has been provided. (Lines 671-700).

- Recommendations for future research are somewhat broad (e.g., promoting mother-daughter communication).

**Response:** Thank you for this suggestion. Please see lines (Lines 694-700; 712-724; 735-739; 756-760).

- While the findings are insightful, practical applications for healthcare providers, educators, or policymakers are not sufficiently detailed. Provide actionable recommendations for interventions, such as educational campaigns, clinic-based counselling, or school programs addressing STI prevention.

**Response:** Thank you for this feedback. More detailed research and applications have been further discussed in the Discussion section. (Lines 694-700; 712-724; 735-739; 756-760).
