# Supplementary material for: The theoretical understanding of risk perception and dual-method contraceptive decision-making among Texas adolescent and young adult cisgender females
Source: PLoS One. 2026 Apr 1;21(4):e0343687. doi: 10.1371/journal.pone.0343687 (PMC13042665; doi:10.1371/journal.pone.0343687)
Supplement: Attachment — Submitted filename: Response to Reviewers_PLOSOne_5November2025 (1).docx [file pone.0343687.s006.docx]

Response to Reviewers

Dear editors of *PLOS ONE,*

Thank you for the opportunity to revise our manuscript, “The Theoretical Understanding of Risk Perception and Dual-Method Contraceptive Decision-Making among Texas Adolescent and Young Adult Women” (PONE-D-24-49168).

We have provided responses to each of the questions from the two reviewers below.

We are happy to provide any additional information that may be needed.

Sincerely,

Lauren Holt

| **Reviewer 1 Feedback** | **Response** |
| --- | --- |
| “The impact of abortion laws and sex education is inferred from self-reports and may not necessarily capture broader societal trends. The authors should clarify that these are perceptions rather than measured behavioral outcomes.” | Thank you for this helpful feedback. Clarification has been provided in the Limitations section (pp. 31, 577-579). |
| “The description of coding lacks detail about how the two analysts resolved discrepancies and whether coding decisions were audited or validated beyond mutual discussion.” | Thank you for this helpful feedback. This information has been provided in the Data Analysis section (pp. 13, lines 187-190). |
| “A further suggestion would be that since maternal influences and partner dynamics emerged as critical elements in the study's findings, the authors could consider highlighting these as recommended avenues for intervention, for example, mother-daughter workshops or developing partner communication models. The authors could also consider discussing policy and clinical implications more explicitly (e.g., the need for sexual health education curricular reforms, or the development of targeted strategies for counseling)” | Thank you for his helpful feedback. These suggestions have been implemented in the Discussion section (pp.28, 509-511; pp. 29-30; 531-533; 546-552) |
|  | |
| **Reviewer 2 Feedback** | **Response** |
| “Condom is already a dual protection  method. It needs to be considered  throughout the work.” | Thank you for this feedback. This matter is addressed on pp. 3; Lines 20-21. |
| “Does it mean that condom not prescribed  for anybody? Why?” | Thank you for this feedback. Given condoms are not prescribed but are part of robust contraceptive counseling sessions, and the focus on participants perception of risk/associated condom use, condoms specifically were not mentioned as part of the inclusion criteria |
| “Why female condom use was  not assessed?” | Thank you for raising this point. For this study, we wanted to focus on any/all condom use, regardless of the type of condom used (male or female). Since both types of condoms prevent against STIs and HIV, we did not find it necessary to differentiate between the two. |
| “I want to know whether one health clinic  can cover all people or just a limited area?” | Thank you for this feedback. The clinic is available to all adolescents and young adults living residing in the large urban county. This has now been specified in the Methods section (pp. 8, 113-120) |
| “If it covers a limited area, then whether it  can be representative for that district?” | Thank you for this feedback. More data has been provided regarding the demographics of the county and the area the clinic resides (pp.8, 113-120) |
| “Which types of social/ economic/ political  features were prominent in the clinic area?” | Thank you for this feedback. The social, economic, and political features of the county the clinic serves have been provided. (pp. 8, 113-117) |
| “It is not clear. I think it needs rewriting.” | Thank you for finding this error. The sentence has been edited for clarity (pp. 20, 332). |
| “It is not clear. Needs rewriting.” | Thank you for this feedback. The sentence has been edited for clarity. (pp.23; Line 393-394). |
| “The sequence of the quotes doesn’t guide  the reader to think about the logic.” | Thank you for this feedback. Edits have been made to differentiate between the two paragraphs and quotes (pp.23, 403) |
| “These two highlighted part has conflict and reject the other one.” | Thank you for this helpful feedback. The two highlighted parts have been amended for consistency. (pp.25; Lines 430-431) |
| “I don’t remember anything about lifelong commitment” | Thank you for this feedback. Lifelong commitment has been removed for consistency. (pp. 27; Line 481) |
| “It is necessary to address HIV differently, as it is not a short-term disease.” | Thank you for this helpful feedback. HIV and other long-term diseases have been addressed. (pp. 28-29; Lines 523-525) |
| “Prior to the provision of free testing, people should be informed that it can’t be an alternative for prevention.” | Thank you for this feedback. This suggestion has been incorporated in the Discussion section (pp. 29, 531-533) |
| “The discussed sexual relation is an  interaction between a male and female.  Interview with some male was necessary.” | Thank you for this feedback. This suggestion has been included in the Limitations section. (pp. 31, 574-577) |
