# Supplementary material for: The theoretical understanding of risk perception and dual-method contraceptive decision-making among Texas adolescent and young adult cisgender females
Source: PLoS One. 2026 Apr 1;21(4):e0343687. doi: 10.1371/journal.pone.0343687 (PMC13042665; doi:10.1371/journal.pone.0343687)
Supplement: Attachment — Submitted filename: Response to Reviewers_6February2026.docx [file pone.0343687.s007.docx]

Response to Reviewers

Dear editors of *PLOS ONE,*

Thank you for the opportunity to revise our manuscript, “The Theoretical Understanding of Risk Perception and Dual-Method Contraceptive Decision-Making among Texas Adolescent and Young Adult Women” (PONE-D-24-49168).

We have provided responses to each of the questions from the two reviewers below.

We are happy to provide any additional information that may be needed.

Sincerely,

Lauren Holt

| **Reviewer 1 Feedback** | **Response** |
| --- | --- |
| “The authors have been responsive to the reviewers’ feedback, and, in its current form, the manuscript is broadly suitable for publication pending only minor editorial polishing by the journal (mainly style and copy-editing). Minor issues that the journal editors can readily address and which do not warrant another round of peer review include occasional typographical spacing or duplicated words (e.g., “effective effective” on page 3).” | Thank you for your feedback. The duplicated “effective” has been removed (pp. 3 Line 17) We appreciate your guidance in revising this manuscript. |
| **Reviewer 2 Feedback** | **Response** |
| “Looking at condom as a dual protection method should be considered throughout of the text. It is not satisfactory to consider it in a limited part of the paper.” | Thank you for this feedback. The research team has more specifically identified condoms as a contraceptive method that can prevent pregnancy, STIs, and HIV when used correctly and consistently, while providing a more in-depth definition of dual-method contraceptive use that specifically focuses on the implementation of, both, a prescribed contraceptive method (the most effective contraceptive method at reducing pregnancy risk) and a condom (the only contraceptive method that can reduce STI and HIV risk) during sexual intercourse. Please see these changes on pages 3-4, lines 19-31 |
| “Contraceptive method selection is based on  a checklist.  Based on some question, client will decid...” | We are unable to read this feedback in its entirety. This feedback cuts off after “client will decide…” Please provide the full feedback in written decision letter. |
| “It will be good to have a look on WHO  guidelines like MEC.” | The WHO lists LARCs as the [most effective prescribed contraceptive method](https://cdn.who.int/media/docs/default-source/reproductive-health/contraception-family-planning/mechanisms-of-action-and-effectiveness-of-contraception-methods.pdf?sfvrsn=e39a69c2_1), but does not explicitly promote LARCs as their first-line recommendation. To incorporate the WHO, we cited their document on LARC (and other contraceptive) effectiveness on pp. 4 Line 38). |
| “Type of sexual relationship is among the  checklist questions.” | Thank you for this feedback. Please see p. 5, line 57-58 |
| “So, it seems family planning guidelines are  not the main reason for increase of LARC  use.” | Thank you for this feedback. We’ve included abortion policy with family planning guidelines (p.4 Lines 38-41). Abortion policy is discussed further on p. 6 lines 74-80. |
| “Based on this text, rules and regulation  changes, especially for abortion, had a  significant impact in this regard.” | Thank you for this feedback. We’ve included abortion policy with family planning guidelines (p.4 Lines 38-41). Abortion policy is discussed further on pp. 6 lines 74-80. |
| “Working on the false rumor is important too.” | Thank you for this feedback. We’ve included these misconceptions on p. 32 Lines 593-601. |
