# Supplementary material for: The theoretical understanding of risk perception and dual-method contraceptive decision-making among Texas adolescent and young adult cisgender females
Source: PLoS One. 2026 Apr 1;21(4):e0343687. doi: 10.1371/journal.pone.0343687 (PMC13042665; doi:10.1371/journal.pone.0343687)
Supplement: Attachment — Submitted filename: Reviewer comments PLOSONE.docx [file pone.0343687.s002.docx]

**Title:** The Theoretical Understanding of Risk Perception and Dual-Method Contraceptive Decision-Making among Texas Adolescent and Young Adult Cisgender Females

Thank you for an opportunity to review the above-mentioned manuscript.

**Reviewer Comments**

**Abstract:** The abstract should be able to “standalone”. The methods sections is inadequate. How many participants were included? Sampling method and data analysis?

**Background**

The manuscript addresses a highly significant public health issue—STIs, HIV risk, and contraceptive use—which is a pressing concern in Texas and nationally. It provides a valuable and timely exploration of STI and HIV risk, contraceptive use, and disparities among AYACF. However, it would benefit from reducing redundancy, clarifying its focus, integrating sections more cohesively, and providing clear research objectives. Addressing these weaknesses will enhance its impact and readability.

- Certain statements, such as the role of risk perception in dual-method contraceptive use, are repeated excessively (e.g., paragraph 4 and paragraph 6).
- The section on *Roe v. Wade* and the subsequent shift in contraceptive behaviours, while relevant, feels somewhat disconnected from the core discussion of STI/HIV risk and dual-method contraceptive use.
- The manuscript attempts to cover a wide range of topics (e.g., STI/HIV disparities, social norms, education policies, healthcare access, relationship dynamics) but risks diluting its focus. Consider narrowing the scope to prioritize the most critical factors affecting STI and HIV risk and dual method contraceptive use.
- There is a mention of "societal and cultural norms and values also play an integral role," it does not sufficiently elaborate on how these norms directly shape decision-making processes for AYACF. Provide specific examples or data illustrating how societal expectations (e.g., stigma around condom use or contraception) influence behaviour.
- While statistics are valuable, the manuscript sometimes lacks contextualization. For example, stating that "23% of AYACF use condoms during every sexual encounter" could benefit from a comparison to other demographic groups or historical trends to underscore the significance of this figure. Supplement raw statistics with interpretative commentary to help the reader understand their implications.
- The gaps in understanding STI and HIV risk perception and dual-method contraceptive decision-making are highlighted, but there is no clear articulation of the research questions or hypotheses driving this investigation. End the introduction with a clear statement of purpose, objectives, or research questions.
- Certain sentences are overly complex, making them harder to follow (e.g., "The existing age, gender, racial, and ethnic STI and HIV disparities highlight the need to consider how social determinants influence dual-method contraceptive use among AYACF").
- The manuscript effectively identifies disparities and contributing factors but lacks a preliminary discussion of potential interventions or strategies to address the issues highlighted.

**Methods**

- The methods align well with the stated aims, especially in exploring AYACF's STI and HIV risk perception and decision-making regarding dual-method contraceptive use. The use of constructivist grounded theory is appropriate for generating middle-range theories from lived experiences.
- There is a mention of “study purpose” in 4.1.2 but then the researchers discuss the objectives of the study instead. There is also “aim 1 and 2”, it is not clear why there is more than one aim. Please revise.
- Include more detail about how reflexivity was actively used to enhance study quality would strengthen the discussion.
- While theoretical sampling is appropriate, the manuscript does not justify why 25 participants were sufficient to achieve saturation. A brief discussion of how saturation was determined could enhance transparency.
- Recruiting participants in person at a health clinic or through referrals may limit the diversity of perspectives, as it excludes AYACF who do not access clinic services. This could bias findings toward individuals with better access to care.
- Although interview questions were developed by a research team specializing in sexual and reproductive health, the manuscript does not specify if pilot testing or validation of the interview guide was conducted before data collection. This omission raises concerns about the reliability of the guide.
- The addition of questions after the 14th interview is described as part of the triangulation process, but it is unclear how this was justified methodologically or whether these changes introduced inconsistencies across interviews.
- While consent is mentioned, there is no explicit discussion of how ethical approval was obtained, how participant confidentiality was maintained (beyond encrypted recording), or how potentially sensitive topics (e.g., relationship violence) were addressed.
- Given the emphasis on racial and ethnic diversity, the methods lack detail on how interviews accounted for cultural sensitivity or if interviewers were trained in cultural competence
- Although NVivo is mentioned as a tool for analysis, there is no discussion of how codes were generated, refined, or validated by the research team. Including examples of codebooks or inter-rater reliability assessments would bolster methodological rigor.
- Include a discussion on how data saturation was determined and whether 25 interviews were sufficient to explore diverse experiences thoroughly.
- Acknowledge potential biases in recruitment and discuss their implications for the study findings.
- Describe the process of pilot testing or validating the interview guide to ensure its reliability and relevance.
- Elaborate on measures taken to ensure cultural competence in interviews and analysis, especially given the focus on racial and ethnic disparities.
- Provide details on ethical approval, how participant confidentiality was maintained, and how sensitive topics were handled.
- Include a description of the coding process, how codes were refined, and how consensus was reached among researchers.

**Results**

- Table 2 contains discrepancies between the written description of demographics and the tabulated data. For instance, racial/ethnic breakdowns in Table 2 do not match those described in the text (e.g., White: 40% vs. 4%; African American: 56% vs. 44%).
- The study focuses on AYACF, but the results do not address potential selection biases or whether findings are generalizable beyond this group.
- Discuss recruitment strategies and limitations related to the sample size (n=25) and its representativeness of broader populations.
- The transition from STI diagnosis to behavior change is not fully unpacked. While some participants report positive changes, others do not, leading to ambiguity about the consistency of these behavioral shifts.
- While quotes provide depth, there is limited quantitative analysis (e.g., patterns or statistical trends across subgroups).
- Expand on how societal expectations about gender influence condom negotiation and decision-making.
- The text mentions "Figure 1: Dual Method Contraception Decision-Making Factors," but the figure is absent.
- Some quotes reiterate similar points (e.g., fear of pregnancy, partner trustworthiness) without adding new insights. Consolidate repetitive examples and focus on unique perspectives to avoid redundancy.

**Discussion**

- Emphasize the diversity in AYACF experiences and caution against generalizing findings to all young adults, particularly those from different socioeconomic or cultural backgrounds.
- Statements like "mother-daughter communication reduces STI and unintended pregnancy" imply causation, which cannot be established from cross-sectional data. Reframe such conclusions to reflect correlations, e.g., "Our findings suggest a potential link between mother-daughter communication and reduced STI and unintended pregnancy risk."
- While some limitations are acknowledged, others are overlooked, such as potential biases in self-reported data and the influence of clinic recruitment settings. Expand the limitations section to address self-reporting bias, recall bias, and the specific impact of recruiting participants from clinics offering free services.
- The discussion mentions societal influences like abortion policies and abstinence-based sex education but does not explore their interplay in detail.
- While the study involves racially and ethnically diverse participants, there is little discussion of how intersecting factors (e.g., race, ethnicity, socioeconomic status) shape their experiences.
- Incorporate an intersectional analysis to enrich the understanding of how various identity factors influence decision-making and risk perception.
- Recommendations for future research are somewhat broad (e.g., promoting mother-daughter communication).
- While the findings are insightful, practical applications for healthcare providers, educators, or policymakers are not sufficiently detailed. Provide actionable recommendations for interventions, such as educational campaigns, clinic-based counseling, or school programs addressing STI prevention.
- Expand on why trust and STI testing as reassurance are prevalent among AYACF. Are these influenced by cultural norms, perceived stigma, or misinformation about STIs?
- Use direct quotes or examples from participants to illustrate key findings, which would provide richer insights and increase engagement.
